# Targeting the CTBP1–CETP axis overcomes ferroptosis resistance in non‐small cell lung cancer by altering lipid accumulation

**DOI:** 10.1002/ctm2.70749

**Published:** 2026-07-27

**Authors:** Yanjie Chen, Heng Wang, Ximin Tan, Chenxi Yan, Fangfang Liu, Shuxuan Deng, Chengyan Wang, Yangchen Xia, Zhaolin Xu, Kongming Wu, Shanshan Huang, Qian Chu

**Affiliations:** ^1^ Department of Oncology Tongji Hospital Tongji Medical College Huazhong University of Science and Technology Wuhan China; ^2^ Department of Thoracic Surgery Tongji Hospital Tongji Medical College Huazhong University of Science and Technology Wuhan China; ^3^ Department of Pathology Shihezi University School of Medicine The First Affiliated Hospital Shihezi University Xinjiang China; ^4^ Cancer Center Tongji Hospital Tongji Medical College Huazhong University of Science and Technology Wuhan China; ^5^ Cancer Center Shanxi Bethune Hospital Shanxi Academy of Medical Science Tongji Shanxi Hospital Third Hospital of Shanxi Medical University Taiyuan China

**Keywords:** CETP, CTBP1, ferroptosis, lipid accumulation, non‐small cell lung cancer, obicetrapib

## Abstract

**Background:**

Non‐small cell lung cancer (NSCLC) remains a leading cause of global cancer mortality. Increasing evidence implicates aberrant cholesterol metabolic reprogramming as a key facilitator of tumour malignancy; however, the mechanistic connections between lipoprotein metabolism and NSCLC pathogenesis remain elusive. Here, we investigate the unrecognised oncogenic role of cholesteryl ester transfer protein (CETP), a central lipid exchange mediator.

**Methods:**

Serum lipid profiles from 151 NSCLC patients were analysed, and CETP mRNA expression level was evaluated in normal lung (*n* = 47) and NSCLC tissue (*n* = 54) and paired NSCLC tissue microarrays (*n* = 113). Integrated ChIP‐seq/RNA‐seq analyses, ChIP–qPCR, dual‐luciferase reporter and ubiquitination assays were performed to investigate CTBP1‐mediated CETP transcription. Functional studies in NSCLC cells, xenograft models and eight patient‐derived organoids evaluated the role of the CTBP1–CETP axis in lipid remodelling, ferroptosis and the therapeutic efficacy of obicetrapib alone or combined with RSL3.

**Results:**

CETP was significantly up‐regulated in NSCLC tissues and predicted poor overall survival in both LUAD (HR = 1.46, 95% CI 1.13–1.87, *p* = .0033) and LUSC (HR = 1.51, 95% CI 1.12–2.01, *p* = .0067). CTBP1 activated CETP transcription, and CTBP1 ubiquitination further enhanced its transcriptional activity. The CTBP1–CETP axis promoted ferroptosis resistance by lipid accumulation. Obicetrapib phenocopied CETP depletion and synergised with RSL3 to inhibit tumour growth in NSCLC cells, xenografts and patient‐derived organoids.

**Conclusions:**

Our study delineates a novel CTBP1–CETP–lipid droplet–MAPK signalling cascade that couples metabolic rewiring with ferroptosis evasion. These insights establish the CTBP1–CETP axis as a critical cell fate determinant, positioning pharmacological CETP inhibition (e.g., obicetrapib) as a translatable therapeutic vulnerability in lipid‐dependent, ferroptosis‐resistant NSCLC.

## Graphical abstract

CTBP1‐driven CETP activation orchestrates lipid accumulation to suppress MAPK‐dependent ferroptosis in non‐small cell lung cancer. Repurposing the clinical‐stage CETP inhibitor obicetrapib successfully overcomes this resistance in NSCLC patient‐derived organoids, highlighting a promising translational therapeutic strategy.

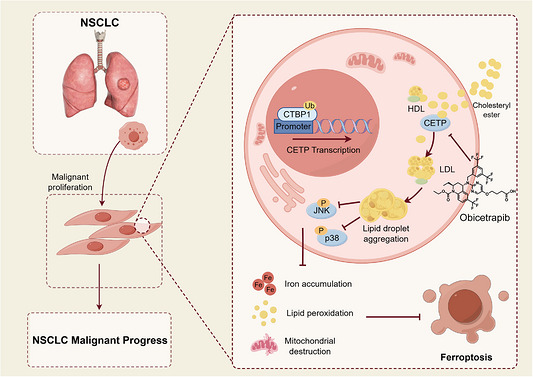

## 1 INTRODUCTION

Lung cancer remains the leading cause of cancer incidence and cancer‐related mortality worldwide. According to the latest data estimates, there were approximately 2.5 million new lung cancer cases globally (12.4% of all cancer diagnoses) and 1.8 million deaths (18.7% of all cancer deaths).^1^ The disease landscape is dominated by non‐small cell lung cancer (NSCLC) ^1^, ^2^, ^3^, ^4^, which accounts for approximately 85% of total diagnoses. Despite therapeutic advancements, NSCLC remains responsible for nearly 80% of lung cancer‐specific deaths, representing a formidable challenge to global public health. ^5^ Prognostic outcomes of NSCLC are highly contingent upon pathological staging at diagnosis. While stage IA NSCLC yields a 5‐year survival rate as high as 82%, this figure exhibits a precipitous decline to a mere 7% in advanced stage IVB cases.^6^

Tumour cells frequently exhibit a hyperactivated cholesterol metabolic profile, characterised by enhanced exogenous uptake and up‐regulated de novo biosynthesis.^7^ This metabolic adaptation furnishes cancer cells with essential building blocks for membrane biogenesis, intracellular signalling intermediates and antioxidant defences.^8^, ^9^ Notably, dysregulated cholesterol metabolism is increasingly recognised as a driver of malignant progression, contributing to tumour proliferation, invasion, immune evasion and therapy resistance.^10^, ^11^ Cholesterol homeostasis is primarily maintained through two lipoprotein‐mediated transport pathways: low‐density lipoprotein (LDL) and high‐density lipoprotein (HDL). Under physiological conditions, LDL transports cholesterol from the liver to peripheral tissues through LDL receptor (LDLR)‐mediated endocytosis and apolipoprotein B (ApoB), whereas HDL facilitates reverse cholesterol transport to the liver, predominantly through apolipoprotein A‐I (ApoA‐I).^12^, ^13^ However, this dynamic balance is often disrupted in the tumour microenvironment. Up‐regulation of LDLR has been implicated in breast cancer progression by fuelling cholesteryl ester (CE) accrual. This lipid accumulation stimulates ERK1/2‐dependent epithelial–mesenchymal transition (EMT), thereby conferring a more aggressive, metastatic phenotype to tumour cells.^14^ In NSCLC, clinical data suggest that elevated circulating LDL levels are closely associated with poor prognosis and attenuated therapeutic responsiveness.^15^, ^16^ Moreover, NSCLC cells demonstrate a heightened dependence on cholesterol compared with normal bronchial epithelial cells, while HDL‐mediated cholesterol efflux via ApoA‐I appears to exert a tumour‐suppressive effect.^17^ Despite these findings, the mechanistic role of LDL as a cholesterol donor in NSCLC pathophysiology remains largely unexplored. Particularly, how LDL reshapes the lipid landscape of tumour cells to promote malignancy has yet to be fully elucidated. In this context, CE transfer protein (CETP), a central mediator of lipid exchange between LDL and HDL, may represent a novel regulatory node. CETP binds CEs and facilitates their transfer from HDL to LDL particles, thereby modulating both systemic and intracellular lipid distribution. While CETP has been a long‐standing focus in cardiovascular research—with recent Phase III clinical trials demonstrating that the CETP inhibitor obicetrapib markedly lowers circulating LDL, ApoB and triglyceride (TG) levels—its potential oncological relevance has remained virtually unexplored.^18^, ^19^, ^20^, ^21^, ^22^ The current lack of comprehensive studies addressing CETP's expression profile, regulatory mechanisms and functional roles in tumour contexts such as NSCLC represents a significant knowledge gap. Deciphering CETP's involvement in NSCLC could therefore not only uncover new layers of lipid‐driven oncogenic signalling but also reveal promising therapeutic targets for metabolic intervention in lung cancer.

C‐terminal binding protein 1 (CTBP1), a member of the CTBP protein family that includes its homolog CTBP2, functions predominantly as a transcriptional corepressor.^23^, ^24^ CTBP1 is widely recognised for its involvement in transcriptional repression, regulation of the cell cycle, cell survival and maintenance of mitotic integrity, and it is crucial for the progression of multiple malignancies.^25^, ^26^ Prior studies have shown that CTBP1 represses a range of tumour suppressor genes, including BRCA1, TP53 and CDH1 (E‐cadherin), thereby facilitating oncogenic transformation and tumour advancement.^26^, ^27^, ^28^, ^29^, ^30^ Intriguingly, accumulating evidence suggests that CTBP1 can also function as a transcriptional activator in certain contexts.^23^, ^31^, ^32^, ^33^, ^34^ Sahu et al. found that, in cells undergoing EMT, CTBP1 binds to the promoter regions of several tumour‐associated genes (FBXO32, MMP1, MMP10, HAS2, CXCR1, IGFBP3 and BMP2). By enhancing chromatin accessibility at these promoter sites, CTBP1 facilitates transcriptional activation of these genes.^31^ Nonetheless, the specific genes regulated by CTBP1 and the precise mechanisms through which it promotes tumourigenesis are not yet fully elucidated.

Our results define CETP as a novel transcriptional effector of CTBP1, noting its pronounced up‐regulation within clinical NSCLC cohorts. Furthermore, CETP expression exhibits a strong positive correlation with pathological staging, suggesting its role as a key driver of malignant progression. Through comprehensive gain‐ and loss‐of‐function analyses, we establish CETP as a central regulator of lipid homeostasis and cell survival. By fostering intracellular LDL accumulation and subsequent lipid droplet (LD) formation, CETP suppresses downstream MAPK signalling, which serves to suppress ferroptosis and fuel accelerated tumour cell expansion. Mechanistically, integrated chromatin immunoprecipitation (ChIP)‐seq and RNA‐seq analyses reveal that CTBP1 directly occupies an active promoter region within the CETP promoter, functioning as a transcriptional activator in this context. Importantly, pharmacological inhibition of CETP using obicetrapib mimics the effects of CETP knockdown and exhibits synergistic anti‐tumour activity when combined with ferroptosis inducers. These findings are further validated in conventional 2D NSCLC cell cultures and patient‐derived organoid (PDO) models. Collectively, our study delineates a novel CTBP1–CETP–LDs–MAPK–ferroptosis axis that orchestrates metabolic reprogramming and ferroptosis resistance in NSCLC and provides compelling preclinical rationale for targeting CETP as a metabolic vulnerability in lipid‐dependent, ferroptosis‐resistant lung cancers.

## 2 MATERIALS AND METHODS

### 2.1 Human NSCLC tissues and clinical data

Surgical specimens from 54 patients with NSCLC and 47 corresponding paratumoural tissues were procured for nucleic acid extraction. This clinical cohort served as the foundation for our comparative molecular analyses and expression profiling (Table **S1**). Additionally, 113 paired NSCLC tumour and adjacent non‐tumourous tissues were obtained to construct tissue microarrays (TMAs) for immunohistochemical analysis (Table **S2**). Tumour and paratumoural tissues were sourced from surgical cases at Tongji Hospital, with histological classification verified through independent double‐blind review by two pathologists. To correlate molecular findings with systemic metabolism, we further integrated clinical lipidomic data from an expanded cohort of 151 NSCLC patients (Table **S3**). Quantification of serum lipid parameters—including total cholesterol (TC), TG and HDL and LDL cholesterol (HDL‐C and LDL‐C, respectively)—were collected within 30 days before and after clinical diagnosis of NSCLC, and all measurements were completed prior to the initiation of any anti‐tumour therapy. All participants provided prior written informed consent in strict accordance with the institutional ethics committee's guidelines. The study protocol was approved by the Institutional Review Board of Ethics Committee of Tongji Hospital affiliated to Tongji Medical College of Huazhong University of Science and Technology (Approval No:TJ‐IRB202603074), and all procedures were conducted in accordance with the Declaration of Helsinki.

### 2.2 Cell lines and cell culture

The human NSCLC cell lines A549 and H1299 were sourced from the Chinese Academy of Sciences (Institute of Biochemistry and Cell Biology, Shanghai). Identity was rigorously authenticated through short tandem repeat profiling (GenePrint® 10 System; Promega) and cross‐referenced with ATCC benchmarks. Cell populations were maintained in RPMI‐1640 medium (Gibco) enriched with 10% heat‐inactivated foetal bovine serum and 1% penicillin–streptomycin (Solarbio) within a humidified 5% CO_2_ environment at 37°C.

### 2.3 NSCLC cell treatments

NSCLC cells were exposed to obicetrapib (MedChemExpress, China) or RSL3 (MedChemExpress) at concentrations equivalent to 50% of their respective IC_50_ values, as determined in preliminary dose–response assays. Cells were treated for 24 h prior to downstream functional analyses. For experiments involving ferroptosis inhibition, A549 and H1299 cells were pre‐treated with ferrostatin‐1 (Fer‐1) (MedChemExpress) at 2 µM. Pre‐treatment durations were 14 h for A549 cells and 18 h for H1299 cells.^35^, ^36^ after which cells were subjected to further treatments, including obicetrapib and RSL3 at 1/2 IC_50_, for subsequent cell assays. All compounds were dissolved in DMSO (MedChemExpress), and vehicle‐treated cells receiving an equivalent concentration of DMSO (<.1%, v/v) were used as controls.

NSCLC cells were supplemented with cholesteryl linoleate (CL) (MedChemExpress), a major CE component of LDL,^37^ to restore LDL‐derived lipid metabolites following CETP knockdown. Cells were treated with 50 µM CL for 24 h before subsequent analyses. The concentration was selected based on previous reports and preliminary optimisation experiments to achieve efficient lipid supplementation under standard culture conditions without affecting cell viability^37^ Cells were then subjected to ferroptosis‐related assays, including C11‐BODIPY staining, Fe^2+^ and malondialdehyde (MDA) measurements, as well as western blotting.

To evaluate the functional role of JNK/p38 MAPK signalling in CETP‐mediated ferroptosis, NSCLC cells were treated with the JNK inhibitor SP600125 (10 µM) or the p38 MAPK inhibitor SB203580 (10 µM) for 12 or 24 h. The inhibitor concentrations were selected based on previous studies demonstrating effective inhibition of JNK and p38 MAPK signalling under comparable experimental conditions^38^, ^39^, ^40^ Following treatment, cells were subjected to immunoblotting, as well as ferroptosis‐related assays, including C11‐BODIPY staining, Fe^2^^+^ and MDA measurements, as well as western blotting.

### 2.4 Plasmids, si/shRNA, cell transfection and lentiviral infection

Full‐length CETP and CTBP1 sequences were integrated into pcDNA3.1 vectors to facilitate gain‐of‐function studies (GeneCreate, China). These expression constructs were generated via PCR‐based amplification and directionally cloned, providing a robust platform for investigating the downstream effects of the CTBP1–CETP axis. A genomic fragment of approximately 1000 bp containing the CTBP1‐binding motif within the CETP promoter was inserted into the pGL3‐promoter luciferase reporter plasmid (Promega, USA).

To silence CTBP1 and CETP expression, specific small interfering RNAs (siRNAs) were designed (GeneCreate) and transfected into cells using Lipo3000 (Thermo Fisher, USA). The sequences are listed in Table **S4**. Cells were collected 24–48 h post‐transfection and subjected to downstream expression analyses and functional experiments.

A FBXO32 deletion mutant lacking amino acids 217–332 (FBXO32‐Δ217–332) was generated using the pcDNA3.1 expression vector. The deleted region was selected based on domain prediction using the InterPro database. All recombinant plasmids were confirmed by Sanger sequencing before subsequent experiments.

For lentiviral‐mediated gene manipulation, CETP and CTBP1 CDS were cloned into the Ubc–MCS–3FLAG–CBh–gcGFP–IRES–puromycin vector (GV492), with the corresponding empty vector serving as a control. For stable knockdown, shRNA sequences targeting CETP and CTBP1 (Table **S4**) were inserted into the hU6–MCS–CBh–gcGFP–IRES–puromycin vector (GV493). Both GV492 and GV493 were obtained from GeneChem (Shanghai, China). To facilitate long‐term functional studies, we established stable CTBP1‐ and CETP‐knockdown NSCLC cells via lentiviral transduction. Recombinant lentiviruses were packaged in HEK293T cells utilising psPAX2 and pMD2.G components. After 48‐h of viral production, viral supernatants were filtered and applied to target cells. Successive puromycin selection (2 µg/mL) was maintained until resistant colonies were established, ensuring a homogeneous population for downstream metabolic and phenotypic assays.

### 2.5 Animal models and husbandry conditions

BALB/c nude mice (4–5 weeks old) served as the in vivo model for this study. Experimental groups were established using a randomised allocation strategy to account for potential confounding variables, with groups matched for both age and biological sex. Housing was provided under specific pathogen‐free conditions, and all protocols were performed in accordance with ethical standards for oncological research.

To assess the in vivo impact of the CTBP1–CETP axis, 1 × 10^7^ A549 cells harbouring either specific shRNAs or non‐targeting controls were subcutaneously inoculated into the right flanks of BALB/c nude mice. Tumour progression was longitudinally monitored at 3‐day intervals via digital caliper measurements. Tumour burden was quantified using the ellipsoidal volume formula, *V* = (length × width^2^)/2, and data are presented as mean ± standard error of the mean (SEM).

For drug treatment xenograft studies, wild‐type A549 cells (1 × 10^7^ cells) were subcutaneously injected in the same manner. Once xenografts reached a mean volume of approximately 100 mm^3^, mice were randomised into cohorts. Standardised caliper measurements were utilised to monitor the subsequent therapeutic response, as detailed in the pharmacodynamic protocols below.

Experimental animal procedures were conducted under the oversight of the Tongji Hospital Animal Ethics Committee (Approval ID: T1‐202509042). Our methodology complied with all relevant ethical regulations and guidelines for animal research, ensuring the highest standards of laboratory animal welfare throughout the study.

### 2.6 Tumour growth and treatments

Obicetrapib (MedChemExpress) was administered orally to mice at a dose of 5 µg/g (5 mg/kg) once daily. To clarify, the dosage of 5 mg/kg used in our mouse model was determined based on the body surface area (BSA) normalisation method, as recommended by United States Food and Drug Administration guidelines. The clinically relevant dose of obicetrapib is 10 mg/day^22^ which equates to approximately .167 mg/kg for a 60 kg human. Using the conversion factor (Km) ratio of 37 (human) to 3 (mouse), the human equivalent dose is calculated as: 5 mg/kg (mouse) × (3/37) ≈ .405 mg/kg (human). This dose (5 mg/kg in mice) corresponds to approximately 24.3 mg/day in humans. We opted for this slightly higher dose (compared with the 10 mg cardiovascular dose) to ensure consistent drug saturation within the tumour microenvironment and to account for the higher metabolic rate of tumour‐bearing mice.

Treatment was initiated on Day 9 (tumours volumes ≈ 100 mm^3^) following tumour cell inoculation and continued throughout the experimental period. A vehicle control group received equivalent volumes of DMSO (MedChemExpress) following the same dosing schedule.

For ferroptosis induction, mice were administered RSL3 (MedChemExpress), a ferroptosis inducer, or DMSO as a vehicle control (MedChemExpress) via intraperitoneal injection beginning 9 days (tumours volumes ≈ 100 mm^3^) after tumour cell implantation. RSL3 was administered at a dose of 5 µg/g (5 mg/kg) every 48 h, and this therapeutic regimen was maintained throughout the study duration to ensure persistent continuous induction of ferroptosis and lipid peroxidation.

### 2.7 RNA extraction and quantitative real‐time PCR

Total RNA from cells and tissues was extracted using TRIzol reagent (Takara, Japan). 1 µg of RNA was reverse‐transcribed into cDNA (Takara). Quantitative PCR (qPCR) analyses were then performed with TB Green® Premix Ex Taq™ II (Takara) on a real‐time PCR (RT‐PCR) platform. Target gene levels were normalised against the housekeeping gene GAPDH to ensure comparative accuracy across experimental groups. All primer sequences can be found in Table **S4**.

### 2.8 Western blot

Cells were lysed under chilled conditions in the presence of protease inhibitors. Supernatants were collected after high‐speed centrifugation to remove insoluble debris, and total protein was quantified via BCA analysis. Protein samples were separated by size using 10% SDS‐PAGE and then immobilised onto PVDF membranes for subsequent immunodetection. Membranes were blocked in TBST (.1% Tween‐20 in TBS) containing 5% non‐fat milk for 1 h at room temperature, then incubated overnight at 4°C with primary antibodies against CTBP1 (10972‐1‐AP; Proteintech; 1:1000), CETP (A1355; ABclonal; 1:1000), ACSL4 (61331; GenuIN; 1:2000), ubiquitin (M026378; Abmart; 1:2000), GAPDH (60004‐1‐Ig; Proteintech; 1:1000), p38 (A5049; ABclonal; 1:2000), p‐p38 (28796‐1‐AP; Proteintech; 1:2000), JNK (66210‐1‐Ig; Proteintech; 1:3000), p‐JNK (AP0631; ABclonal; 1:2000), GPX4 (67763‐1‐Ig; Proteintech; 1:2000). Protein–antibody complexes were labelled with secondary HRP‐conjugated IgG (Proteintech) and detected through ECL‐based fluorography (Abbkine).

### 2.9 Immunohistochemistry

Following deparaffinisation and heat‐mediated antigen recovery, tissue slides were blocked in 10% goat serum (37°C, 30 min). Subsequently, sections were probed with primary antibodies against CTBP1 (10972‐1‐AP; Proteintech; 1:100), CETP (A1355; ABclonal; 1:100), GPX4 (67763‐1‐Ig; Proteintech; 1:100), 4‐hydroxynonenal (4‐HNE) (PC6313; Abmart; 1:200) and Ki67 (ER1706‐46; HUABIO; 1:200) at 4°C for 16–18 h. The following day, slides were incubated with biotinylated secondary antibodies (45 min, 25°C) and visualised using a DAB chromogen kit (ZSGB‐BIO; ZLI‐9018). After haematoxylin counterstaining, sections were dehydrated and mounted for microscopic evaluation. The immunohistochemistry (IHC) staining results were independently and blindly evaluated by two experienced pathologists. A semi‐quantitative scoring system was implemented based on both the percentage of positive cells and the staining intensity. The percentage of positive tumour cells was scored on a scale of 0 to 4: 0, 0%; 1, 1–10%; 2, 11–50%; 3, 51–75%; and 4, >75%. The staining intensity was graded on a scale of 0 to 3: 0, no staining; 1, weak staining; 2, moderate staining; and 3, strong staining. The final immunoreactivity score for each specimen was determined by multiplying the positive percentage score by the intensity score, yielding a final range of 0 to 12. Any divergent assessments between the two pathologists were resolved through re‐evaluation and mutual consensus.

### 2.10 Cell proliferation and colony formation assays

A549 or H1299 cells were seeded at a density of 1500 cells/well in 96‐well plates. At 24 h intervals (up to 96 h), 10 µL of CCK‐8 reagent was added per well followed by a 2‐h incubation. Absorbance was subsequently recorded to evaluate growth kinetics. NSCLC cells from each treatment group were seeded at 1500 cells per well in six‐well plates with medium replaced every 3 days. When individual colonies contained more than 50 cells, they were fixed with 4% paraformaldehyde, stained with crystal violet and manually counted under a light microscope.

### 2.11 Cell migration and invasion assays

Cell motility and invasiveness were evaluated using Transwell chambers (Corning Falcon, USA). For invasion assays, inserts were pre‐coated with Matrigel, whereas migration was assessed using uncoated membranes. To quantify cellular transit, NSCLC cells (A549 (6 × 10^4^), H1299 (8 × 10^4^)) were placed in the primary compartment of the Transwell. A nutrient gradient (10% FBS) was utilised to stimulate transmigration over a 24‐h period. Subsequently, translocated cells were stained with crystal violet and quantified across three stochastic fields to ensure statistical representativeness.

### 2.12 ChIP and qPCR

We performed ChIP analysis utilising reagents from ABclonal (China). Cells were first cross‐linked and lysed, and the chromatin was fragmented by sonication. Immunoprecipitation was carried out overnight at 4°C using either an anti‐CTBP1 antibody (22008‐1‐AP; Proteintech), anti‐H3K4me3 antibody (22146; ABclonal) or control rabbit IgG (RM20712; ABclonal), with protein A/G magnetic beads employed to capture the complexes. The DNA fractions were isolated utilising a spin‐column‐based purification methodology (ABclonal, China).

Enrichment of specific DNA sequences was assessed by qPCR (primers listed in Table S4). For visualisation, ChIP–qPCR products were resolved on a 2% agarose gel. Electrophoresis was conducted in TAE buffer at 100 V for 30–40 min, and DNA bands were detected and photographed under UV illumination.

### 2.13 Luciferase reporter assay

The activity of luciferase reporters was evaluated using the Dual‐Luciferase Reporter Kit (Vazyme, China). Reporter constructs containing the ∼1‐kb CETP promoter region (inclusive of the CTBP1‐binding motif) were introduced into cells. To ensure internal calibration, pRL‐SV40 (Promega) was co‐transfected, facilitating the dual‐luciferase analysis of promoter activity. Following transfection, luminescence was recorded by luminometer. Firefly luciferase data were normalised to Renilla luciferase signals to correct for differences in transfection efficiency.

### 2.14 Iron assay

The ferrous iron (Fe^2^^+^) concentration in NSCLC cells was determined using the Iron Assay Kit (Thermo Fisher Scientific) according to the manufacturer's instructions. Cells were lysed in iron assay buffer and incubated on ice for .5 h. Following centrifugation at 12 000×*g* for 10 min, the supernatant was transferred to a 96‐well plate and incubated with 50 µL of the iron‐detecting probe at 25°C for 60 min. The absorbance was then measured at 593 nm using a microplate reader.

### 2.15 MDA assay

MDA levels in NSCLC cells were determined using the MDA Assay Kit (Solarbio, China). Cell samples were first homogenised and combined with the supplied reaction reagent. The reaction mixtures were subjected to thermal incubation at 95°C for 1–2 h to allow MDA to react with thiobarbituric acid (TBA), generating a pink MDA–TBA complex. Samples were centrifuged to remove any precipitates, and the supernatant was loaded in a 96‐well plate. Lipid peroxidation levels were determined by measuring at 532 nm. The MDA values were adjusted for protein concentration (determined by BCA assay).

### 2.16 LDL‐C assay

LDL‐C levels in cell lysates were quantified using the LDL‐C Assay Kit (Yeasen, China). Samples were mixed thoroughly with the provided reaction reagent and incubated at 37°C for 10 min to allow for enzymatic reactions specific to LDL‐C. After incubation, absorbance was measured at 600 nm using a microplate reader. LDL‐C concentrations were determined based on a standard curve and expressed in mmol/L or mg/dL.

### 2.17 BODIPY 493/503 staining for LD detection

Intracellular LD accumulation was assessed using a lipid droplet green fluorescence detection kit (Beyotime Biotechnology, Shanghai, China; Cat. No. C2053S). After experimental treatments, the culture medium was removed, and the cells were gently washed twice with PBS. Working solutions of 1× BODIPY 493/503 and 1× Hoechst 33342 were prepared according to the manufacturer's instructions and used to stain LDs and nuclei, respectively. After incubation at 37°C for 30 min in the dark, the cells were washed twice with PBS to remove excess dye. BODIPY 493/503 fluorescence was observed under a fluorescence microscope using an excitation wavelength of approximately 488 nm. Fluorescence intensity in each group was quantified using ImageJ software.

### 2.18 Lipid peroxidation detection

The level of intracellular lipid peroxidation levels were measured by the lipid peroxidation assay kit with BODIPY™ 581/591 C11 (Beyotime Biotechnology). Freshly BODIPY 581/591 C11 was prepared following the manufacturer's guidelines and added to the wells. Cells were then incubated at 37°C in the dark for .5 h. Excess dye was removed by washing the cells twice with PBS, and fluorescence was either visualised under a fluorescence microscope (Ex/Em = 581/589 nm) or quantified by flow cytometry.

For lipid peroxidation analysis in organoid samples, the lipid peroxidation assay kit with BODIPY™ 581/591 C11 (Beyotime Biotechnology) was used. Organoids were loaded in freshly prepared BODIPY 581/591 C11 working solution at 37°C for half hour in the dark. Fluorescence was captured using a fluorescence microscope, where the non‐oxidised probe was detected at Ex/Em = 581/589 nm, and the oxidised lipid peroxidation products were detected at 488/510 nm.

### 2.19 RNA sequencing and analysis

mRNA libraries were generated by Novogene (Beijing, China) following standard procedures. Library fragments were amplified via PCR and size‐selected to enrich inserts between 350 and 550 bp. Sequencing was performed on the Illumina Xplus platform with 150 bp paired‐end reads. Raw reads were processed to remove adapters and low‐quality sequences to generate clean reads, which were then aligned to the Homo sapiens GENCODE GRCh38.v35 reference genome using STAR (v2.7.8a). Transcriptomic read counts were generated using featureCounts (v2.0.3) and analysed for differential expression via the DESeq2 R package.

### 2.20 ChIP sequencing and analysis

ChIP‐seq experiments in NSCLC cell lines were performed following established protocols. The purified DNA was then utilised to construct ChIP‐seq libraries, which were prepared by Novogene Corporation.

Raw FASTQ reads were filtered using fastp (v0.19.11) to remove adapters and low‐quality sequences, generating clean reads with calculated Q20, Q30 and GC content metrics. Filtered reads were aligned to the Homo sapiens GENCODE GRCh38.v35 reference genome using BWA‐MEM (v0.7.12).

Peak identification was carried out using MACS2 (v2.1.0), comparing IP samples against the input control, with a *q*‐value cutoff of .05 applied to define significant enrichment. Motif discovery and enrichment analyses were performed using HOMER (v4.10). Functional annotation of genes associated with ChIP‐seq peaks was conducted via Gene Ontology (GO) and Kyoto Encyclopedia of Genes and Genomes (KEGG) enrichment analyses using KOBAS (v2.1.1), and statistically significant enrichment was defined as corrected *p* values < .05. The raw ChIP‐seq datasets have been successfully uploaded to the ZENODO (https://doi.org/10.5281/zenodo.20829734).

### 2.21 Organoid models

NSCLC tissues were collected from patients and processed for the establishment of organoid cultures. Freshly resected NSCLC tumour tissues were washed with cold PBS containing antibiotics. The tissue were enzymatically digested in a solution containing collagenase type II (1 g/L) and DNase I (100 U/mL) at 37°C for half hour with gentle agitation. After digestion, the resulting cells were resuspended in cold Matrigel (Corning Falcon) and seeded as 30 µL domes in pre‐warmed 24‐well plates. After Matrigel solidification at 37°C for 10–15 min, organoid culture medium was added to each well. The organoid cultures were maintained under optimised growth conditions, and their morphological features and size were assessed by using bright‐field microscopy. Comprehensive information regarding patient specimen sources, clinical background and sample sizes for the PDOs has been compiled and added to Table **S5**.

### 2.22 Organoid models‐drug treatments

NSCLC‐derived organoids were cultured in Matrigel‐based 3D conditions according to standard protocols. Drug concentrations were selected with reference to the effective concentrations established in the two NSCLC cell lines. Unless otherwise indicated for dose–response experiments, organoids were treated with 10 µM obicetrapib for 7 days. To evaluate organoid growth and viability, organoids were exposed to increasing concentrations of obicetrapib, either alone or in combination with 2.5 µM RSL3, for 7 days. For ferroptosis rescue experiments, organoids were pre‐treated with 2 µM Fer‐1 for 18 h prior to the addition of dose‐escalating obicetrapib and 2.5 µM RSL3. Following treatments, organoid growth and cell viability measurement using CellTiter‐Glo 3D (Promega). All experiments were performed with at least three independent organoid cultures to ensure reproducibility.

### 2.23 Statistical analysis

Statistical analyses were carried out using GraphPad Prism (v10.4), SPSS (v21.0) or R (v3.3.0). Data normality was rigorously evaluated using the Shapiro–Wilk test prior to secondary inferential statistics. Data are presented as mean ± standard deviation (SD). Statistical comparisons between two groups were performed using a two‐tailed Student's *t*‐test, with unpaired or paired analyses applied according to the experimental design. For analyses involving more than two groups, one‐way analysis of variance (ANOVA) was performed as appropriate, followed by Tukey's post hoc multiple‐comparison test. Tumour growth curves and cell proliferation curves measured over time were analysed using two‐way repeated‐measures ANOVA. Spearman's correlation analysis was employed to assess gene co‐expression relationships. Kaplan–Meier survival curves were generated to evaluate overall survival in NSCLC patients, and statistical significance was determined using the log‐rank test.

All in vitro experiments included three technical replicates and were independently repeated in at least three biological replicates. A *p* value of less than .05 was considered statistically significant. In figure legends, statistical significance is indicated as: *p < .05, **p < .01, ***p < .001, while non‐significant differences are labelled as ‘NS’.

## 3 RESULTS

### 3.1 Elevated CETP expression in NSCLC is associated with poor prognosis

**FIGURE 1:**
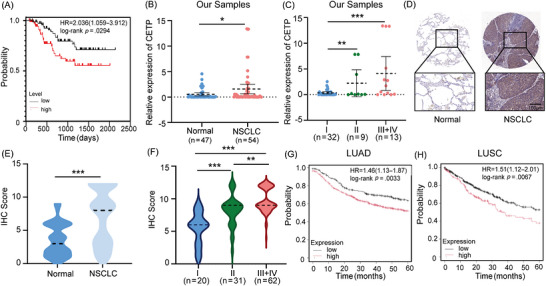
Elevated expression of CETP in NSCLC predicts poor prognosis. (A) Kaplan–Meier overall survival (OS) analysis of 151 NSCLC patients stratified by serum LDL‐C levels. (B) Relative RNA levels of CETP in NSCLC tumour tissues (*n* = 54) and adjacent normal tissues (*n* = 47). (C) CETP expression levels stratified by clinical stage, Stage I (*n* = 32), Stage II (*n* = 9), Stage III and IV (*n* = 13). (D) Representative immunohistochemical staining of CETP in NSCLC tissue microarrays and quantification of CETP protein expression. Scale bar, 100 µm. (E) The quantitative analysis of CETP protein expression in NSCLC tissue microarrays assessed by IHC staining. (F) Comparison of CETP protein levels across clinical stages. (G and H) Kaplan–Meier OS of lung adenocarcinoma (LUAD) (*n* = 906) and lung squamous cell carcinoma (LUSC) (*n* = 495) from the KMplotter database. All data represent mean ± SD from at least three independent experiments. *p < .05, **p < .01, ***p < .001.

Given that previous studies have demonstrated a significant association between elevated circulating LDL levels and poor prognosis as well as diminished therapeutic response in NSCLC.^15^, ^16^ we first analysed serum lipid profiles from a cohort of 151 NSCLC patients, including measurements of TC, TG, LDL‐C and HDL‐C. In line with previous reports, survival analysis revealed that elevated LDL‐C levels were associated with reduced overall survival in these patients (Figure **1A** **)**. In contrast, TC, TG and HDL‐C showed no significant associations with survival (Figure **S1A–C**). To further explore molecular mechanisms underlying this observation, we examined CETP expression in paired clinical NSCLC specimens. RT‐qPCR revealed a significant up‐regulation of CETP mRNA in tumour tissues relative to adjacent normal tissues (Figure **1B**). Moreover, stratification by clinical stage revealed significantly higher CETP expression in tumours from patients with advanced disease relative to those with early‐stage NSCLC (Figure **1C**). At the protein level, immunohistochemical analysis of TMAs confirmed stronger CETP staining in tumour tissues compared with adjacent non‐tumour counterparts (Figure 1D,E), with expression levels again enriched in advanced‐stage cases (Figure **1F**). Finally, independent validation using the KMplotter online database further demonstrated that high CETP expression was consistently associated with poor prognosis in both lung adenocarcinoma and squamous cell carcinoma (Figure 1G,H).

### 3.2 CETP drives tumourigenesis in NSCLC by regulating lipid accumulation

**FIGURE 2:**
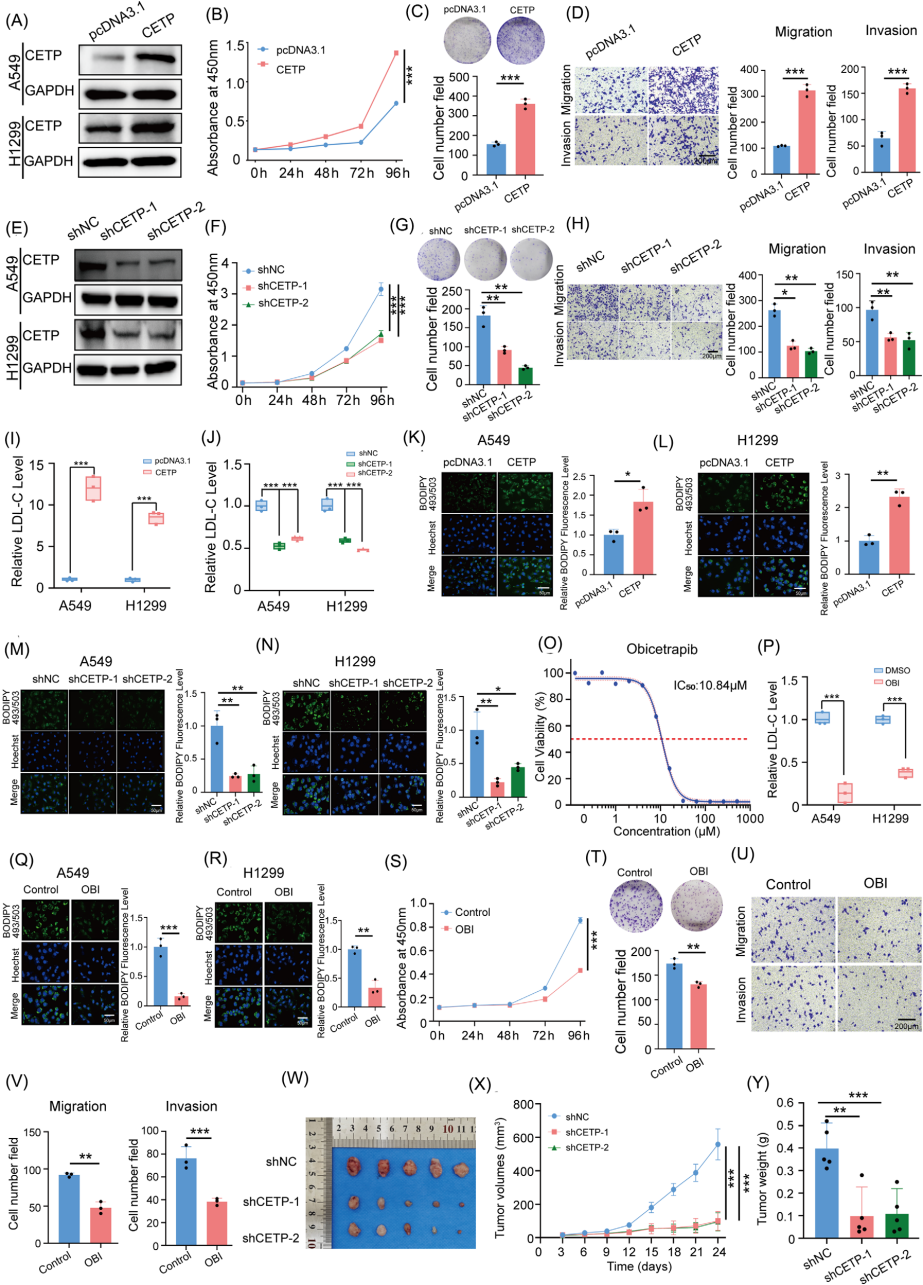
CETP drives tumourigenesis in NSCLC by regulating lipid accumulation. (A) Western blot analysis showing CETP protein levels in A549 and H1299 cells following CETP overexpression. (B) Cell growth curves of A549 cells with or without CETP overexpression by CCK‐8 assays. (C) CETP contributes to the proliferation of A549 cells as measured by colony formation assays. (D) Representative images of migration and invasion assays in CETP‐overexpressing A549 cells. Scale bar, 200 µm. (E) Western blot validation of CETP knockdown efficiency in NSCLC cells generated via lentiviral transduction. (F) Effects of CETP knockdown on abilities of A549 cells’ proliferation. (G) Colony formation assays were performed in A549 cells upon CETP knockdown. (H) Transwell assays detecting migration and invasion of A549 cells with CETP depletion. Scale bar, 200 µm. (I and J) Quantification of intracellular LDL‐C levels in A549 and H1299 cells following CETP overexpression (I) or knockdown (J). (K and L) Representative BODIPY 493/503 fluorescence images and quantitative analysis of neutral lipid accumulation in A549 (K) and H1299 (L) cells following CETP overexpression. (M and N) Representative BODIPY 493/503 fluorescence images and quantitative analysis of neutral lipid accumulation in A549 (M) and H1299 (N) cells following CETP knockdown. (O) Determination of the IC_50_ of obicetrapib in A549 cells. (P) Quantification of intracellular LDL‐C levels in A549 and H1299 cells following treatment with obicetrapib at 1/2 IC_50_. (Q and R) Representative BODIPY 493/503 fluorescence images and quantitative analysis of neutral lipid accumulation in A549 (Q) and H1299 (R) cells following treatment with obicetrapib at 1/2 IC_50_. (S–V) CCK‐8, colony formation and Transwell migration and invasion assays assessing the effects of obicetrapib at 1/2 IC_50_ on A549 cells. Scale bar, 200 µm. (W–Y) In vivo evaluation of CETP function using a subcutaneous xenograft mouse model, including representative tumour images (W), tumour growth curves (X) and final tumour weights (Y). Data from in vitro experiments represent mean ± SD from three independent experiments. Data from in vivo experiments are presented as mean ± SEM (*n* = 5 mice per group) *p < .05, **p < .01, ***p < .001, NS, not significant.

To evaluate the functional contribution of CETP in NSCLC, we established A549 and H1299 cell lines with CETP overexpression (Figures 2A and S2A). The impact of CETP up‐regulation on cell proliferation, migration and invasion was subsequently examined. Results from both CCK‐8 and colony formation assays revealed that CETP overexpression markedly enhanced NSCLC cell growth (Figures 2B,C and S2B,C), supporting its oncogenic activity in NSCLC. Furthermore, Transwell assays revealed that CETP overexpression dramatically increased the migratory and invasive capacities of NSCLC cells (Figures 2D and S2D). To further confirm the oncogenic role of CETP in NSCLC, A549 and H1299 cells were stably transduced with lentiviruses expressing CETP‐targeting shRNAs (Figures 2E and S2E). Consistent with expectations, CETP depletion significantly inhibited the proliferation, migration and invasion capacities of both A549 and H1299 cells (Figures 2F–H and S2F–H). Given that CETP is a CE transfer protein responsible for shuttling CE to LDL, thereby promoting CE accumulation.^18^, ^19^, ^20^ we therefore measured intracellular LDL‐C levels in NSCLC cells following CETP overexpression or knockdown as a readout of CE accumulation. Overexpression of CETP led to a significant increase in intracellular LDL‐C levels in NSCLC cells (Figure 2I). Conversely, CETP knockdown resulted in a concomitant suppression of LDL‐C accumulation (Figure 2J). Consistent with the increased intracellular LDL‐derived lipid accumulation, BODIPY 493/503 staining further demonstrated that CETP overexpression markedly promoted cytoplasmic LD accumulation (Figure 2K,L), whereas CETP knockdown significantly reduced intracellular LD formation (Figure 2M,N). These findings indicate that CETP facilitates the storage of lipids in NSCLC cells. Obicetrapib, a next‐generation highly selective CETP inhibitor that potently suppresses CETP‐mediated lipid transport activity, has been shown in recent Phase III clinical trials to significantly reduce circulating levels of LDL, ApoB and TG.^21^, ^22^ To investigate whether obicetrapib could counteract the oncogenic effects mediated by CETP, we first determined its half‐maximal inhibitory concentration (IC_50_) in A549 and H1299 cells (Figures 2O and S2I). Notably, CETP overexpression significantly increased the sensitivity of NSCLC cells to obicetrapib, as evidenced by a reduction in IC_50_ values in both A549 (2.46 µM) and H1299 (1.39 µM) cells compared with control groups (Figure S2J,K). These results suggest that CETP expression status modulates cellular responsiveness to obicetrapib treatment.

As anticipated, obicetrapib treatment significantly reduced intracellular LDL‐C levels (Figure 2P). Consistently, BODIPY 493/503 staining demonstrated a substantial reduction in intracellular LD accumulation (Figure 2Q,R). To further assess the functional impact of obicetrapib, CCK‐8, colony formation and Transwell assays were conducted using cells treated with obicetrapib at 1/2 of its IC_50_ concentration. Obicetrapib treatment markedly suppressed NSCLC cell growth and proliferative capacity, accompanied by a significant reduction in migratory and invasive abilities (Figures 2S–V and S2L–N). To further validate the role of CETP in NSCLC, we utilised a subcutaneous xenograft tumour model for in vivo studies. Mirroring the in vitro findings, CETP depletion significantly suppressed in vivo tumour growth compared with control groups (Figure 2W–Y). Taken together, these results from both in vitro and in vivo experiments demonstrate that CETP promotes malignant progression in NSCLC.

### 3.3 CTBP1 enhances CETP expression in NSCLC through transcriptional regulation

**FIGURE 3:**
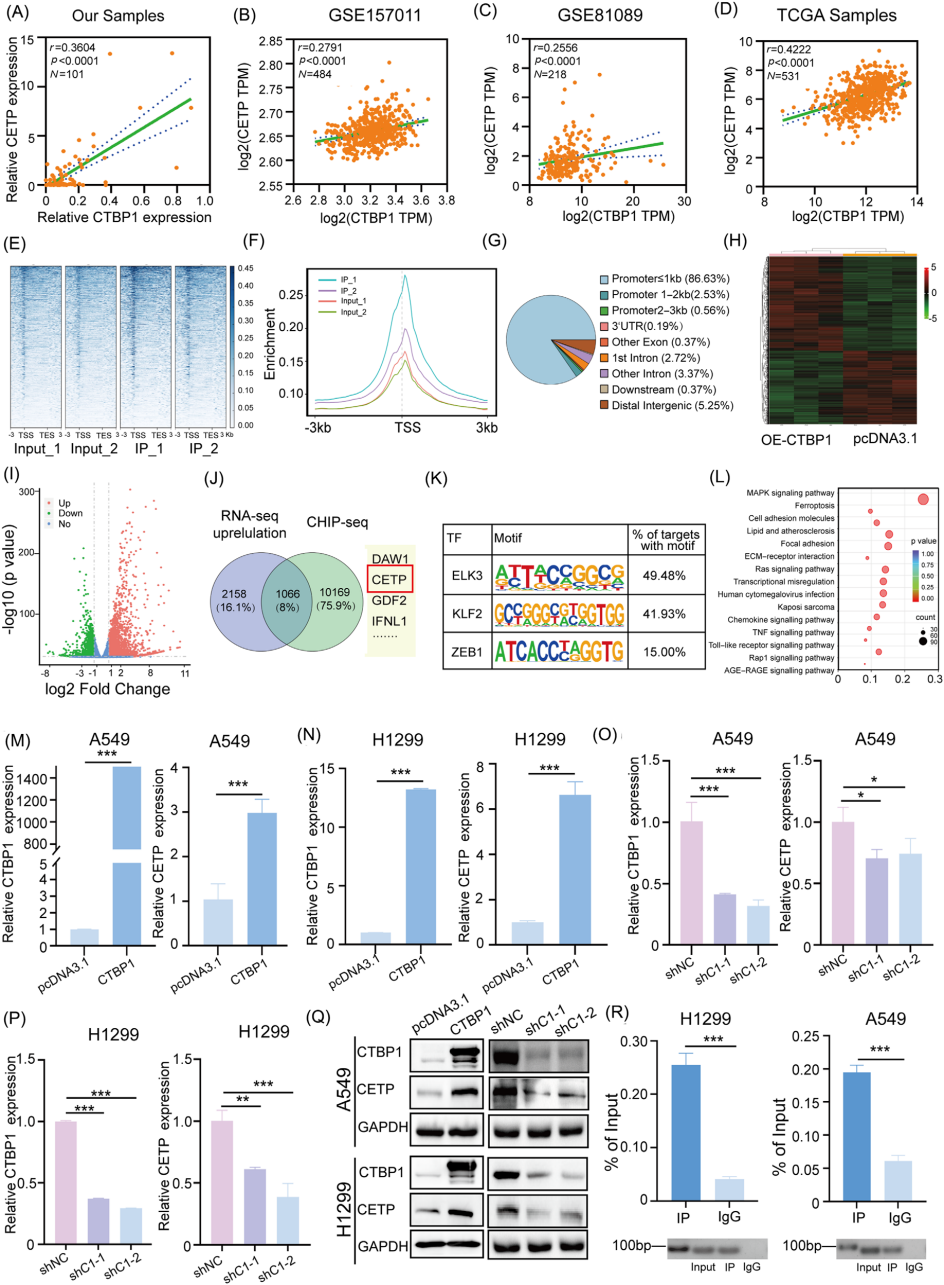
CTBP1 enhances CETP expression in NSCLC through transcriptional regulation. (A–D) Correlation analyses of CTBP1 and CETP expression levels across multiple datasets, including the in‐house NSCLC cohort (A), GEO datasets (B and C) and TCGA database (D). (E) Heatmap showing the CTBP1‐bound peaks in A549 cells. (F) Distribution of CTBP1‐bound and input peaks on genes in A549 cells. (G) Distribution of CTBP1‐bound peaks on different regions across chromatin. (H) Heatmap of differentially expressed genes from RNA‐seq analysis of A549 cells upon CTBP1 overexpression. (I) Volcano plot of CTBP1 overexpression from RNA‐seq analysis in A549 cells. (J) A Venn diagram shows the potential targets of CTBP1 through the combined analysis of RNA‐seq, ChIP‐seq from the present study in A549 cells. (K) Enriched motifs at the promoters of genes upon CTBP1. (L) KEGG pathway enrichment analysis of CTBP1 target genes highlighting pathways associated with MAPK signalling, lipid metabolism and ferroptosis. (M–P) qRT‐PCR analyses showing CETP expression following CTBP1 overexpression (M and N) or knockdown (O and P) in NSCLC cells. (Q) The protein levels of CETP and CTBP1 in NSCLC cells upon CTBP1 overexpression and knockdown were measured. (R) ChIP–PCR validation of CTBP1 binding to the CETP promoter region in A549 and H1299 cells. shC1, shCTBP1. All data represent mean ± SD from at least three independent experiments. *p < .05, **p < .01, ***p < .001, NS, not significant.

To elucidate the molecular basis underlying the aberrant up‐regulation of CETP in NSCLC, we first sought to identify transcriptional regulators that may contribute to its dysregulated expression. Using the GeneCards database, we screened for candidate upstream transcription factors of CETP. To filter these candidates, we evaluated their expression correlation with CETP in the TCGA database and their association with patient prognosis via the KMplotter database. Based on these clinical correlation analyses and literature review regarding oncogenic functions in lung cancer, CTBP1 was selected as a candidate for further investigation. Notably, although CTBP1 is characterised as a transcriptional corepressor, our multi‐omics and preliminary data indicated a positive regulatory relationship with CETP transcription, leading us to investigate this specific transcriptional regulation. To validate this regulatory axis, we performed correlation analyses across multiple independent datasets, including our institutional NSCLC cohort, the GEO database and the TCGA database (Figure 3A–D). CTBP1 and CETP expression levels showed a positive correlation in all datasets, suggesting that CETP activation in NSCLC may, at least in part, be driven by CTBP1.

We next performed ChIP‐seq in A549 cells (Figure 3E). Binding analysis revealed that CTBP1 occupancy was predominantly enriched at gene promoter regions, with over 90% of peaks located within ±3 kb of the transcription start site (TSS) (Figure 3F,G). RNA sequencing following CTBP1 overexpression in A549 cells revealed 3244 up‐regulated genes (Figure 3H‐I). By integrating ChIP‐seq and RNA‐seq datasets, we identified 1066 transcripts, including CETP, that were both bound by CTBP1 and differentially expressed upon its overexpression (Figure 3J). Motif analysis of CTBP1‐binding regions revealed enrichment of consensus motifs corresponding to several lipid metabolism‐related transcription factors, such as ELK3, KLF2 and ZEB1 (Figure 3K).^41^, ^42^, ^43^, ^44^, ^45^, ^46^ Pathway enrichment analysis of these target genes revealed significant enrichment in the MAPK signalling pathway, as well as tumour‐associated pathways such as lipid metabolism and ferroptosis (Figure 3L).

To experimentally validate these findings, we performed qRT‐PCR and western blot analyses. CTBP1 overexpression significantly increased both CETP mRNA and protein levels in NSCLC cells (Figures 3M,N,Q and S4A), whereas CTBP1 inhibition markedly reduced CETP expression at both transcript and protein levels (Figures 3O–Q and S4E). Furthermore, ChIP–PCR assays in A549 and H1299 cells confirmed the genomic binding of CTBP1 to the CETP promoter genomic region (Figure 3R). Collectively, these results demonstrate that CTBP1 functions as a positive transcriptional regulator of CETP, thereby contributing to its oncogenic regulatory network in NSCLC.

### 3.4 CTBP1 facilitates transcriptional up‐regulation by specifically engaging the CETP promoter region

**FIGURE 4:**
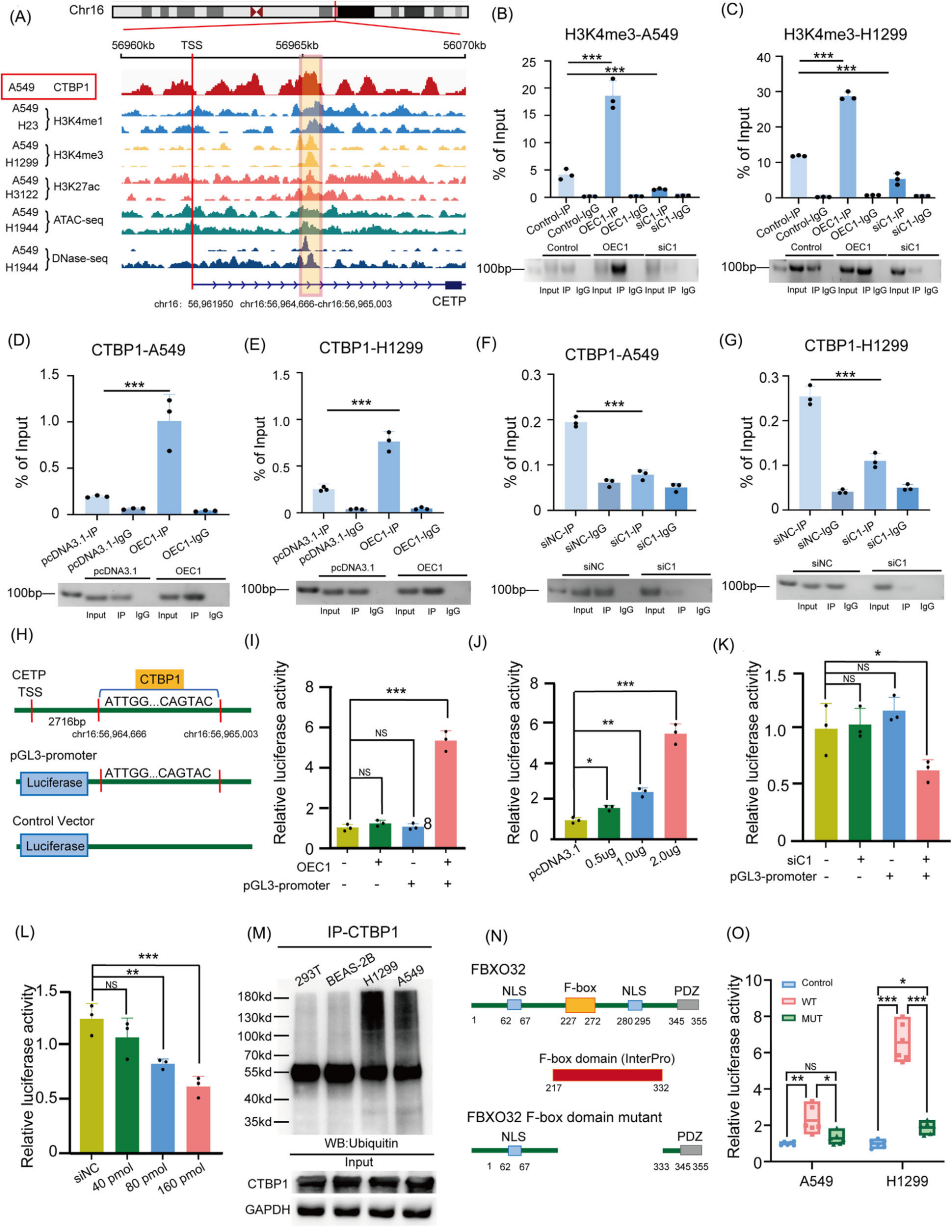
CTBP1 promotes CETP transcription through epigenetic activation of its promoter. (A) Genome browser view showing CTBP1 binding at the CETP promoter region, overlapping with active histone modification marks (H3K4me1, H3K4me3, H3K27ac) and chromatin accessibility signals (DNase‐seq and ATAC‐seq peaks). (B and C) ChIP–PCR analysis of H3K4me3 enrichment at the CETP promoter region following CTBP1 knockdown or overexpression in A549 and H1299 cells. (D–G) ChIP–qPCR detecting the interaction between CTBP1 and the promoters of CETP under CTBP1 knockdown or overexpression conditions in A549 and H1299 cells. (H) Plasmid construction for the dual‐luciferase reporter assay. (I and J) Dual‐luciferase reporter assays showing dose‐dependent increases in CETP promoter activity upon CTBP1 overexpression in A549 cells. (K and L) Dual‐luciferase reporter assays showing reduced CETP promoter activity following siRNA‐mediated CTBP1 knockdown in A549 cells. (M) CTBP1 ubiquitination in 293T, BEAS‐2B, H1299 and A549 cells was assessed by immunoprecipitation using an anti‐CTBP1 antibody followed by immunoblotting with an anti‐ubiquitin antibody. (N) Schematic representation of the full‐length FBXO32 and the FBXO32‐mut deletion mutant, in which the F‐box domain (amino acids 217–332) was deleted. (O) Dual‐luciferase reporter assay was performed to measure the relative luciferase activity of FBXO32 wild‐type and FBXO32‐mut deletion mutant (amino acids 217–332) in A549 and H1299 cells. MUT, FBXO32‐mut deletion mutant; OEC1, overexpression CTBP1; siC1, siCTBP1; WT, FBXO32‐wild type. All data represent mean ± SD from at least three independent experiments. *p < .05, **p < .01, ***p < .001, NS, not significant.

CTBP1 plays a critical role in transcriptional up‐regulation by specifically engaging the CETP promoter region. Previous studies have demonstrated that CTBP1 promotes transcription through the regulation of chromatin accessibility^23^, ^31^, ^32^, ^33^, ^34^ Notably, CTBP1 binds to the CETP promoter region, where it overlaps with several active histone modifications, including H3K4me1, H3K4me3 and H3K27ac, as well as chromatin accessibility marks such as DNase‐seq and ATAC‐seq peaks. This overlap is consistent with a transcriptionally active state (Figure 4A). To further investigate the functional role of CTBP1 in this process, we analysed the enrichment of H3K4me3 modifications in the CETP promoter region by manipulating CTBP1 expression in A549 and H1299 cell lines, both through knockdown and overexpression approaches. ChIP–PCR analysis revealed that in the absence of CTBP1, the enrichment of H3K4me3 modifications on the target sequence was significantly reduced. Conversely, overexpression of CTBP1 led to an increase in the enrichment of this active histone mark (Figure 4B,C). After the overexpression or knockdown of CTBP1, the ChIP–PCR enrichment of CTBP1 protein on the target sequence increased or decreased accordingly after the overexpression or knockdown of CTBP1 (Figure 4D–G **)**. These findings suggest that CTBP1 modulates the H3K4me3 histone modification status at the CETP promoter and TSS regions. To further confirm the regulatory role of CTBP1 in transcriptional activation, we performed dual‐luciferase reporter assays. We cloned the CETP promoter region, identified by ChIP‐seq as a CTBP1‐binding site, into a dual‐luciferase reporter plasmid (Figure 4H). Overexpression of CTBP1 led to a dose‐dependent increase in luciferase activity, demonstrating its positive regulatory effect on transcription (Figures 4I,J and S3A,B). In contrast, siRNA‐mediated knockdown of CTBP1 expression led to a corresponding gradient decrease in transcriptional activity, further supporting the involvement of CTBP1 in transcriptional regulation (Figures 4K,L and S3C,D).

Although CTBP1 has been previously characterised predominantly as a transcriptional corepressor, emerging evidence suggests that its function may be context‐dependent and capable of transcriptional activation under specific cellular conditions. In particular, recent studies have reported that FBXO32 can mediate CTBP1 ubiquitination, thereby modulating its chromatin‐associated behaviour and influencing transcriptional activity through alterations in histone modification patterns^31^ Consistent with these reports, FBXO32 has been shown to be up‐regulated in NSCLC and is associated with poor patient prognosis^47^, ^48^ suggesting a potential functional relevance of FBXO32‐mediated regulation in tumour progression. Based on these observations, we hypothesised that aberrant ubiquitination of CTBP1 may contribute to its altered transcriptional activity in NSCLC. To test this hypothesis, we examined the ubiquitination status of endogenous CTBP1. Immunoprecipitation assays revealed that CTBP1 ubiquitination levels were significantly higher in NSCLC cell lines (A549 and H1299) compared with 293T cells and normal human bronchial epithelial cells (BEAS‐2B) (Figure 4M), indicating enhanced post‐translational modification of CTBP1 in NSCLC. We generated an FBXO32 truncation mutant lacking the F‐box domain.^31^ (115 amino acids, including a conserved 45‐amino‐acid core F‐box region predicted by InterPro) (Figure 4N) and assessed its regulatory effect using dual‐luciferase reporter assays. Wild‐type FBXO32 significantly increased CETP promoter activity, whereas deletion of the F‐box domain markedly weakened its transcriptional promoting effect (Figure 4O **),** indicating that FBXO32‐mediated ubiquitination capacity is required for CTBP1‐dependent transcriptional activation of CETP.

### 3.5 CTBP1 functions as a potential tumour promoter gene in NSCLC

**FIGURE 5:**
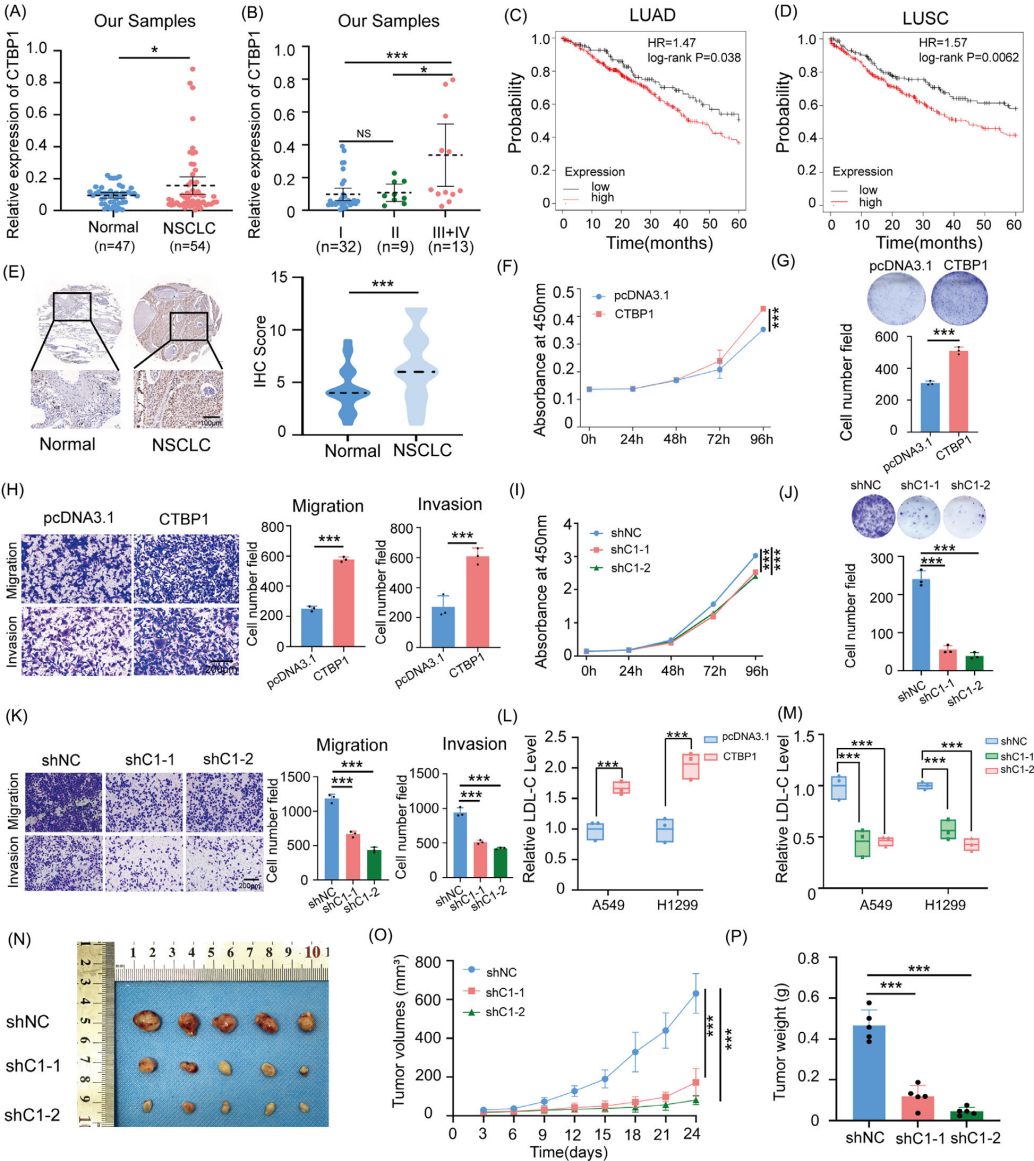
CTBP1 promotes tumourigenesis of NSCLC in vitro and in vivo. (A and B) Relative RNA levels of CTBP1 mRNA expression in NSCLC tissues (*n* = 54) and adjacent normal tissues (*n* = 47), stratified by clinical stage (Stage I (*n* = 32), Stage II (*n* = 9), Stage III and IV (*n* = 13)). (C and D) Kaplan–Meier survival analysis showing overall survival of NSCLC patients with high or low CTBP1 expression in lung adenocarcinoma (*n* = 504) and squamous cell carcinoma (*n* = 495) cohorts. (E) Representative immunohistochemical staining of CTBP1 protein in NSCLC and matched adjacent normal tissues from tissue microarrays. Scale bar, 100 µm. (F–H) CCK‐8, colony formation and Transwell assays showing enhanced proliferation, migration and invasion of A549 cells upon CTBP1 overexpression. Scale bar, 200 µm. (I–K) Inhibition of cell proliferation, migration and invasion following CTBP1 knockdown in A549 cells. Scale bar, 200 µm. (L and M) Measurement of intracellular LDL‐C levels showing increased accumulation in CTBP1‐overexpressing cells and reduction upon CTBP1 silencing in A549 cells. (N–R) Representative images and quantitative analysis of xenograft tumour growth in nude mice injected with CTBP1 knockdown or control NSCLC cells. shC1, shCTBP1. Data from in vitro experiments represent mean ± SD from three independent experiments. Data from in vivo experiments are presented as mean ± SEM (*n* = 5 mice per group). *p < .05, **p < .01, ***p < .001, NS, not significant.

We first performed a comparative analysis of the mRNA expression levels of CTBP1 in NSCLC tissues versus adjacent normal tissues. Analysis showed that CTBP1 expression was markedly elevated in NSCLC tissues, with significantly higher levels observed in advanced‐stage tumours compared with early‐stage cases (Figure 5A,B **)**. Kaplan–Meier survival analysis was performed to evaluate the relationship between CTBP1 expression and patient outcomes in NSCLC. The results indicated that elevated CTBP1 levels were significantly associated with poorer prognosis in both lung adenocarcinoma and squamous cell carcinoma, the two predominant histological subtypes of NSCLC (Figure 5C,D). In addition, analysis of an NSCLC TMA revealed that CTBP1 protein expression was significantly elevated in tumour tissues compared with matched adjacent normal lung tissues (Figure 5E). Collectively, these results reveal that CTBP1 is consistently up‐regulated in NSCLC and associated with poor prognosis. Next, we investigated the functional role of CTBP1 in NSCLC. Overexpression of CTBP1 significantly enhanced cell proliferation, migration and invasion (Figures 5F–H and S4B–D). In contrast, knockdown of CTBP1 notably inhibited these cellular processes in NSCLC cells (Figures 5I–K and S4F–H). As an upstream regulator of CETP, we also assessed the impact of CTBP1 on LDL‐C levels. Overexpression of CTBP1 in A549 and H1299 cells led to a significant accumulation of LDL‐C, whereas knockdown of CTBP1 suppressed this effect (Figure 5L,M). To explore the oncogenic potential of CTBP1 in vivo, we performed subcutaneous tumour formation assays. Compared with the control group, CTBP1 knockdown significantly reduced the tumour volume and weight in xenografted mice (Figure 5N–R). Thus, these results demonstrate that CTBP1 enhances tumour progression of NSCLC and is associated with poor prognosis.

### 3.6 CTBP1–CETP axis regulates ferroptosis and malignant progression in NSCLC

**FIGURE 6:**
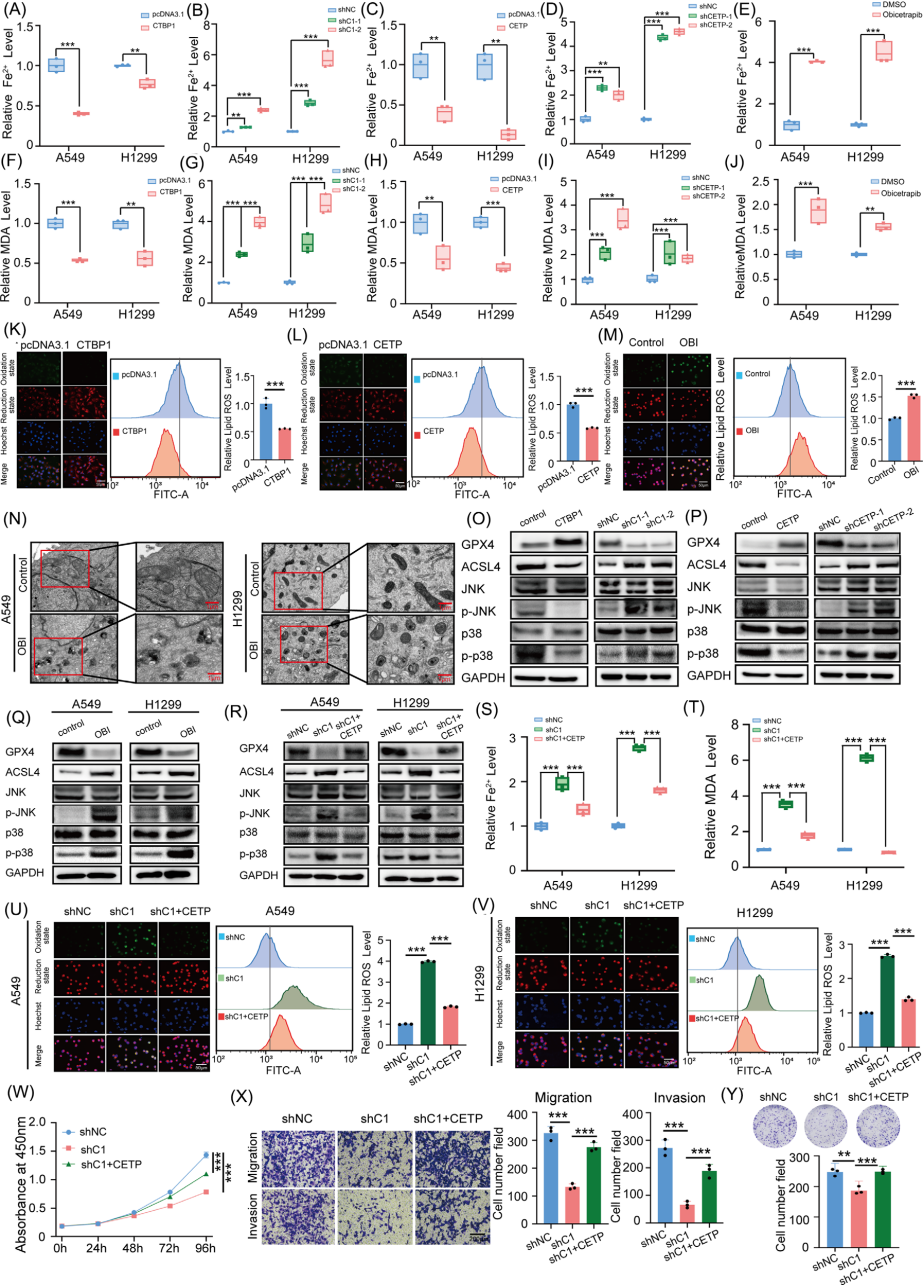
CTBP1–CETP axis modulates MAPK signalling and ferroptosis to promote NSCLC malignancy. (A–E) Quantification of intracellular Fe^2^^+^ levels in NSCLC cells following CTBP1 or CETP overexpression, knockdown or treatment with the CETP inhibitor obicetrapib. (F–J) Quantification of MDA levels in NSCLC cells after CTBP1 or CETP overexpression, knockdown or treatment with the CETP inhibitor obicetrapib. (K–M) Detection of intracellular lipid peroxidation levels by C11‐BODIPY 581/591 staining and flow cytometry in A549 cells under the indicated treatments. Scale bar, 50 µm. (N) Transmission electron microscopy images displaying mitochondrial shrinkage and autophagosome formation characteristic of ferroptosis in obicetrapib‐treated NSCLC cells. Scale bar, 1 µm. (O and P) Western blot analysis of GPX4/ACSL4 expression and JNK/p38 phosphorylation in response to CTBP1/CETP modulation in A549 cells. (Q) Western blot analysis of GPX4/ACSL4 expression and phosphorylation status of JNK and p38 in response to obicetrapib treatment at 1/2 IC_50_. (R) Western blot analysis of GPX4/ACSL4 expression and phosphorylation status of JNK and p38 in NSCLC cells that overexpressed CETP after knockdown of CTBP1. (S) Quantification of intracellular Fe^2+^ levels in NSCLC cells with CTBP1 knockdown followed by CETP overexpression (rescue experiment). (T) Quantification of intracellular MDA levels in NSCLC cells with CTBP1 knockdown followed by CETP overexpression (rescue experiment). (U and V) Detection of intracellular lipid peroxidation levels by C11‐BODIPY 581/591 staining and flow cytometry in A549 and H1299 cells under control, CTBP1 knockdown and CETP rescue conditions. Scale bar, 50 µm. (W–Y) Rescue experiments showing that CETP reintroduction into CTBP1‐knockdown A549 cell reverses the suppression of proliferation, migration and invasion. Scale bar, 200 µm. shC1, shCTBP1. All data represent mean ± SD from at least three independent experiments. *p < .05, **p < .01, ***p < .001, NS, not significant.

Ferroptosis, a type of cell death triggered by iron‐dependent lipid peroxidation, has been increasingly recognised for its involvement in tumour progression, with lipids serving a central role in this process.^49^, ^50^, ^51^ Recently, Calhoon et al. discovered that lipid supplementation might help cancer cells resist ferroptosis, with both LDL and HDL effectively inhibiting lipid peroxidation, although LDL has a more sustained effect.^51^ KEGG enrichment analysis also identified a significant number of genes associated with ferroptosis (Figure 3L). To directly assess ferroptosis‐related changes, intracellular Fe^2^^+^ levels were measured in cells overexpressing CTBP1 or CETP. Compared with the control group, both CTBP1 and CETP overexpression significantly reduced Fe^2^^+^ levels (Figure 6A,C). In contrast, knockdown of CTBP1 or CETP markedly elevated Fe^2^^+^ levels (Figure 6B,D). Treatment with the CETP inhibitor obicetrapib also increased intracellular Fe^2^^+^ levels in NSCLC cells (Figure 6E). We further measured MDA levels. The results showed a significant reduction in MDA levels in cells overexpressing CTBP1 or CETP (Figure 6F,H **)**. Conversely, knockdown of CTBP1 or CETP, as well as treatment with obicetrapib, led to increased MDA levels, further confirming the inhibitory role of the CTBP1–CETP axis in ferroptosis (Figure 6G,I,J). The C11‐BODIPY staining and flow cytometry analyses demonstrated that elevated expression of CTBP1 or CETP significantly reduced NSCLC intracellular lipid peroxidation levels (Figures 6K,L and S5A,B). In contrast, knockdown of CTBP1 or CETP significantly increased A549 and H1299 intracellular lipid peroxidation levels (Figure S5D–G). Furthermore, treatment with obicetrapib also markedly enhanced NSCLC lipid peroxidation (Figures 6M and S5C). Transmission electron microscopy further confirmed the characteristics of ferroptosis, with increased mitochondrial shrinkage and autophagosome formation observed in obicetrapib‐treated NSCLC cells (Figure 6N).

KEGG pathway analysis revealed that the enriched signalling pathways were predominantly clustered in MAPK signalling, with JNK and p38 pathways being most frequently associated with increased lipid ROS accumulation and ferroptosis susceptibility.^52^, ^53^, ^54^, ^55^, ^56^ The results of western blotting revealed that overexpression of CTBP1 increased the ferroptosis‐related protein GPX4 expression while suppressing the ACSL4 expression and phosphorylation of JNK and p38, conversely CTBP1 knockdown reduced GPX4 expression and markedly enhanced ACSL4 expression and JNK/p38 phosphorylation (Figures 6O and S5H,I). Similar results were observed for CETP, consistent with its role as a downstream effector of CTBP1 (Figures 6P and S5J,K). Treatment with the CETP inhibitor obicetrapib also decreased GPX4 protein levels and significantly activated ACSL4 expression and JNK/p38 phosphorylation (Figures 6Q and S5L). Furthermore, to verify the cooperative oncogenic role of CTBP1 and CETP in NSCLC, CETP was overexpressed into CTBP1‐knockdown cells and its impact on GPX4 expression, ACSL4 expression and JNK/p38 signalling was analysed. Restoration of CETP expression not only rescued GPX4 levels and suppressed ACSL4 expression and JNK/p38 phosphorylation but also reversed the inhibitory effects of CTBP1 depletion on NSCLC cell proliferation, migration and invasion (Figures 6R and S5M). Consistently, CTBP1 knockdown‐induced Fe^2+^ accumulation and MDA production were effectively reversed by CETP overexperssion in NSCLC cells (Figure 6S,T). Consistently, CETP re‐expression markedly reversed the increase in lipid peroxidation, as evidenced by C11‐BODIPY staining in both A549 and H1299 cells following CTBP1 knockdown (Figure 6U,V). In addition, CETP restoration significantly attenuated the inhibitory effects of CTBP1 depletion on NSCLC cell proliferation, as well as cell migration and invasion, as demonstrated by CCK‐8, colony formation, wound healing and Transwell assays (Figures 6W–Y and S5N–P). Collectively, these findings demonstrate that the CTBP1–CETP axis suppresses ferroptosis in NSCLC by up‐regulating GPX4 expression and inhibiting ACSL4 expression JNK/p38 signalling, thereby promoting malignant progression.

### 3.7 Rescue experiments confirm CETP as a key regulator of ferroptosis

To determine whether CETP knockdown‐induced cell death was ferroptosis dependent, NSCLC cells with CETP depletion were treated with the ferroptosis inhibitor Fer‐1.

**FIGURE 7:**
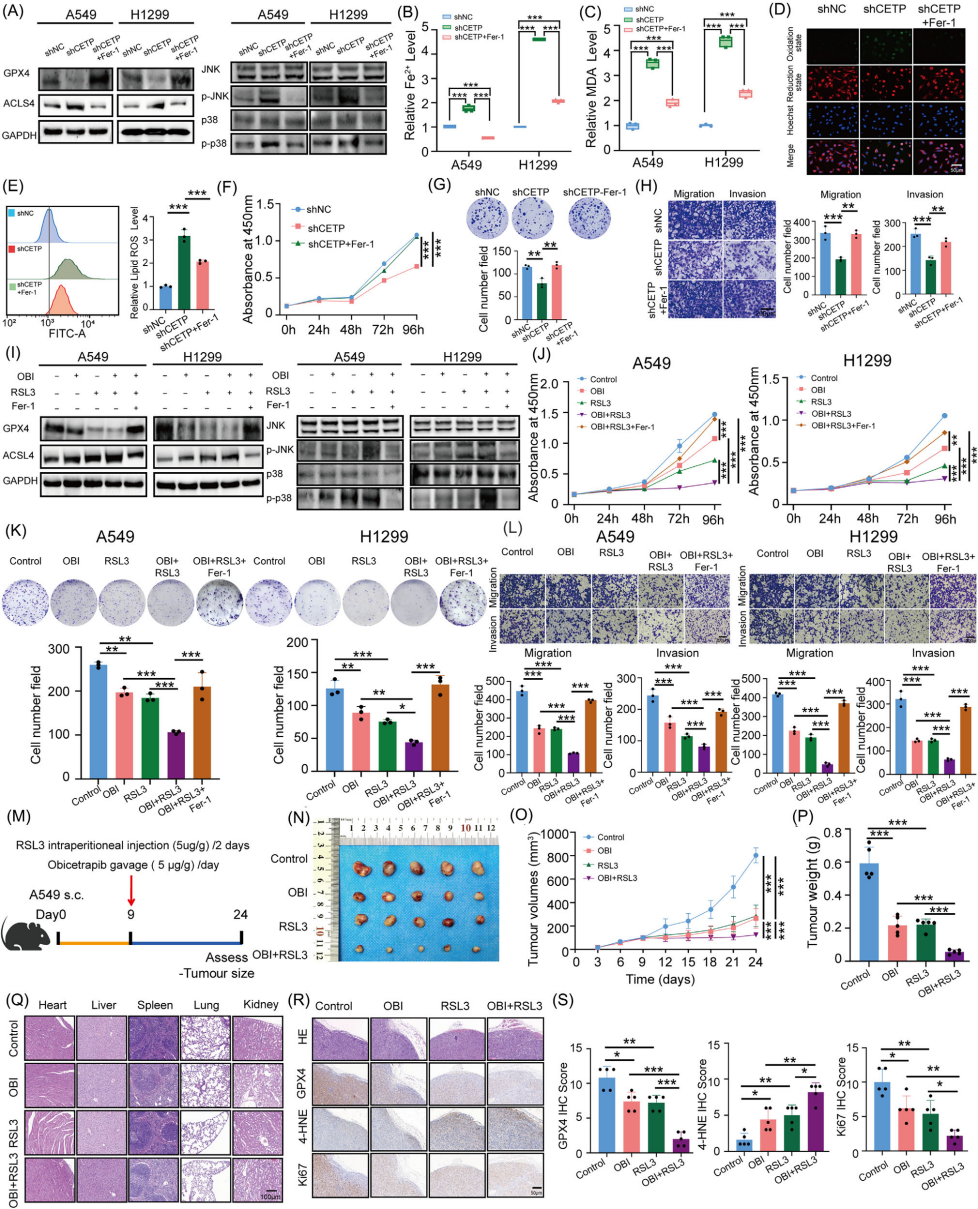
CETP regulates ferroptosis and mediates the therapeutic response to obicetrapib. (A) Western blot analysis of GPX4 and ACSL4 expression and phosphorylation levels of JNK and p38 in CETP‐knockdown NSCLC cells following treatment with the ferroptosis inhibitor Fer‐1. (B) Intracellular Fe^2+^ levels were measured in CETP‐knockdown NSCLC cells following Fer‐1 treatment. (C) MDA levels were measured in CETP‐knockdown NSCLC cells following Fer‐1 treatment. (D) Representative BODIPY 581/591 C11 staining images showing lipid peroxidation in CETP‐knockdown A549 cells following Fer‐1 treatment. Scale bar, 50 µm. (E) Flow cytometric analysis of lipid peroxidation levels in CETP‐knockdown A549 cells following Fer‐1 treatment. (F and G) Cell viability and colony formation assays in CETP‐knockdown A549 cells following Fer‐1 treatment. (H) Transwell migration and invasion assays in CETP‐knockdown A549 cells following Fer‐1 treatment. Scale bar, 200 µm. (I) Western blot analysis of GPX4 and ACSL4 expression and JNK/p38 phosphorylation in NSCLC cells following treatment with obicetrapib (OBI), RSL3, OBI + RSL3 or OBI + RSL3 + Fer‐1. (J and K) CCK‐8 and colony formation assays in NSCLC cells following treatment with obicetrapib, RSL3, OBI + RSL3 or OBI + RSL3 + Fer‐1. (L) Transwell assays in NSCLC cells following treatment with obicetrapib (OBI), RSL3, OBI + RSL3 or OBI + RSL3 + Fer‐1. (M–P) Animal experiment design (M) and representative tumour images (N), volume (O) and weight (P) analysis in each group following OBI, RSL3 or combined treatment. (Q) HE staining of major organs (heart, liver, spleen, lung and kidney) collected from treated mice. Scale bar, 100 µm. (R and S) Immunohistochemical staining of GPX4 and Ki67 in xenograft tumour tissues from each treatment group. Scale bar, 50 µm. Fer‐1, ferrostatin‐1; OBI, obicetrapib. All data represent mean ± SD from at least three independent experiments. *p < .05, **p < .01, ***p < .001, NS, not significant.

At the protein level, Fer‐1 restored GPX4 expression and attenuated ACSL4 up‐regulation as well as JNK/p38 MAPK phosphorylation induced by CETP knockdown, consistent with suppression of ferroptotic signalling (Figures 7A and S6A). Consistently, CETP knockdown‐induced Fe^2^^+^ accumulation and MDA production were effectively reversed by Fer‐1 treatment in NSCLC cells (Figure 7B,C). Lipid peroxidation was further assessed using C11‐BODIPY staining, which showed that Fer‐1 markedly reduced the elevated lipid peroxidation induced by CETP depletion in A549 and H1299 cell lines (Figures 7D,E and S6B). Functionally, Fer‐1 significantly rescued the inhibitory effects of CETP knockdown on NSCLC cell proliferation, migration and invasion (Figures 7F–H and S6C–E). These findings confirm that CETP knockdown induces ferroptosis‐dependent cell death in NSCLC cells.

To further determine whether lipid depletion contributes to CETP knockdown‐induced ferroptosis, NSCLC cells with CETP depletion were supplemented with exogenous CL (LDL‐C component)^37^ as a lipid rescue approach. At the molecular level, LDL‐C supplementation restored GPX4 expression and attenuated ACSL4 up‐regulation in both A549 and H1299 cells (Figure S7A,B). Consistently, Fe^2+^ accumulation and MDA production were also markedly decreased following LDL‐C treatment (Figure S7C,D). Finally, C11‐BODIPY staining demonstrated that LDL‐C supplementation effectively suppressed the elevated lipid peroxidation induced by CETP depletion (Figure S7E,F). Collectively, these results indicate that exogenous lipid replenishment can partially rescue CETP knockdown‐induced ferroptosis by restoring intracellular lipid homeostasis in NSCLC cells.

Given that lipid supplementation only partially reversed CETP knockdown‐induced ferroptotic alterations, we next investigated whether downstream stress signalling pathways were involved in mediating this effect. To this end, NSCLC cells with CETP depletion were treated with the JNK inhibitor SP600125 (Figure S7G,H) or the p38 MAPK inhibitor SB203580 (Figure S7I,J) for 12 and 24 h. Notably, phosphorylation levels of JNK and p38, as well as changes in GPX4 and ACSL4 expression, exhibited a time‐dependent amplification pattern. Specifically, both MAPK activation and ferroptosis‐related marker alterations progressively changed from 12 to 24 h. Pharmacological inhibition of either JNK or p38 signalling significantly attenuated CETP knockdown‐induced ferroptotic changes, as evidenced by decreased intracellular Fe^2+^ accumulation (Figure S7K,L), lower MDA (Figure S7M,N) and levels educed lipid peroxidation (Figure S7O–R). Collectively, these rescue experiments demonstrate that CETP depletion induces ferroptosis in NSCLC cells through coordinated disruption of lipid metabolism and MAPK‐dependent stress signalling, which can be partially reversed by lipid supplementation or pharmacological inhibition of JNK/p38 pathways.

### 3.8 Targeting CETP with obicetrapib improves response to ferroptosis induction in NSCLC

Obicetrapib, a next‐generation CETP inhibitor, has been shown to significantly reduce LDL cholesterol levels and improve cardiovascular outcomes and is currently undergoing large‐scale cardiovascular outcome trials.^20^, ^21^, ^22^ Given the regulatory role of CETP in ferroptosis, we further explored whether obicetrapib could potentiate ferroptosis induction in NSCLC.

The ferroptosis inducer RSL3 exhibited IC_50_ values of 14.03 and 5.05 µM in A549 and H1299 cells, respectively (Figure S6F,G). To evaluate the therapeutic potential of CETP inhibition and its relationship with ferroptosis sensitivity, NSCLC cells were treated with obicetrapib, the ferroptosis inducer RSL3, Fer‐1 or their combinations. Western blot analysis showed that obicetrapib treatment increased ACSL4 and decreased GPX4 expression, whereas RSL3 further biological phenocopied or accelerated these changes, while Fer‐1 reduced these effects in obicetrapib‐ and RSL3‐co‐treated NSCLC cells, confirming ferroptosis dependence (Figures 7I and S6H). Consistent with its role in CETP inhibition, combined treatment with obicetrapib and RSL3, a ferroptosis inducer, markedly suppressed cell proliferation, as demonstrated by cell viability and colony formation assays; this suppression was significantly reversed by Fer‐1, a ferroptosis inhibitor (Figure 7J,K **)**. Similarly, the obicetrapib and RSL3 combination treatment more effectively reduced NSCLC cell migration and invasion than either monotherapy; this suppression was significantly reversed by Fer‐1 (Figure 7L).

Obicetrapib, clinically administered orally at 10 mg/day for cardiovascular disease, was evaluated for its anti‐tumour effects in an NSCLC mouse model, alone or in combination with the ferroptosis inducer RSL3. The in vivo dose of RSL3 was selected based on previous NSCLC mouse studies.^57^ while the dose of obicetrapib was determined via human‐to‐mouse BSA conversion.^22^ with an additional factor accounting for the higher basal metabolic rate of mice, resulting in 5 µg/g body weight administered once daily by oral gavage to mimic clinical usage. Compared with control or single‐agent treatments, the combination of obicetrapib and RSL3 produced the most pronounced inhibition of tumour growth and reduction in tumour weight (Figure 7M–P). Histological analysis of major organs—including the heart, liver, spleen, lung and kidney—using haematoxylin and eosin staining showed no apparent pathological changes in any of the treatment groups relative to controls (Figure 7Q **)**. No significant changes in body weight were observed during the treatment period, indicating minimal systemic toxicity (Figure S6I). Immunohistochemical staining of tumour tissues demonstrated that GPX4, a key ferroptosis marker, and Ki67, a proliferation marker, were both significantly decreased in the combination group compared with other groups, consistent with the in vitro observations. In contrast, 4‐HNE, a marker of lipid peroxidation, was markedly increased in the combination group, further confirming enhanced ferroptotic lipid damage in vivo (Figure 7R,S). Together, these results indicate that pharmacological inhibition of CETP by obicetrapib enhances the therapeutic efficacy of ferroptosis inducers against NSCLC, achieving potent anti‐tumour activity without causing mouse systemic adverse effects.

### 3.9 Organoid models validate the anti‐tumour effect of the CETP inhibitor obicetrapib via ferroptosis regulation in NSCLC

**FIGURE 8:**
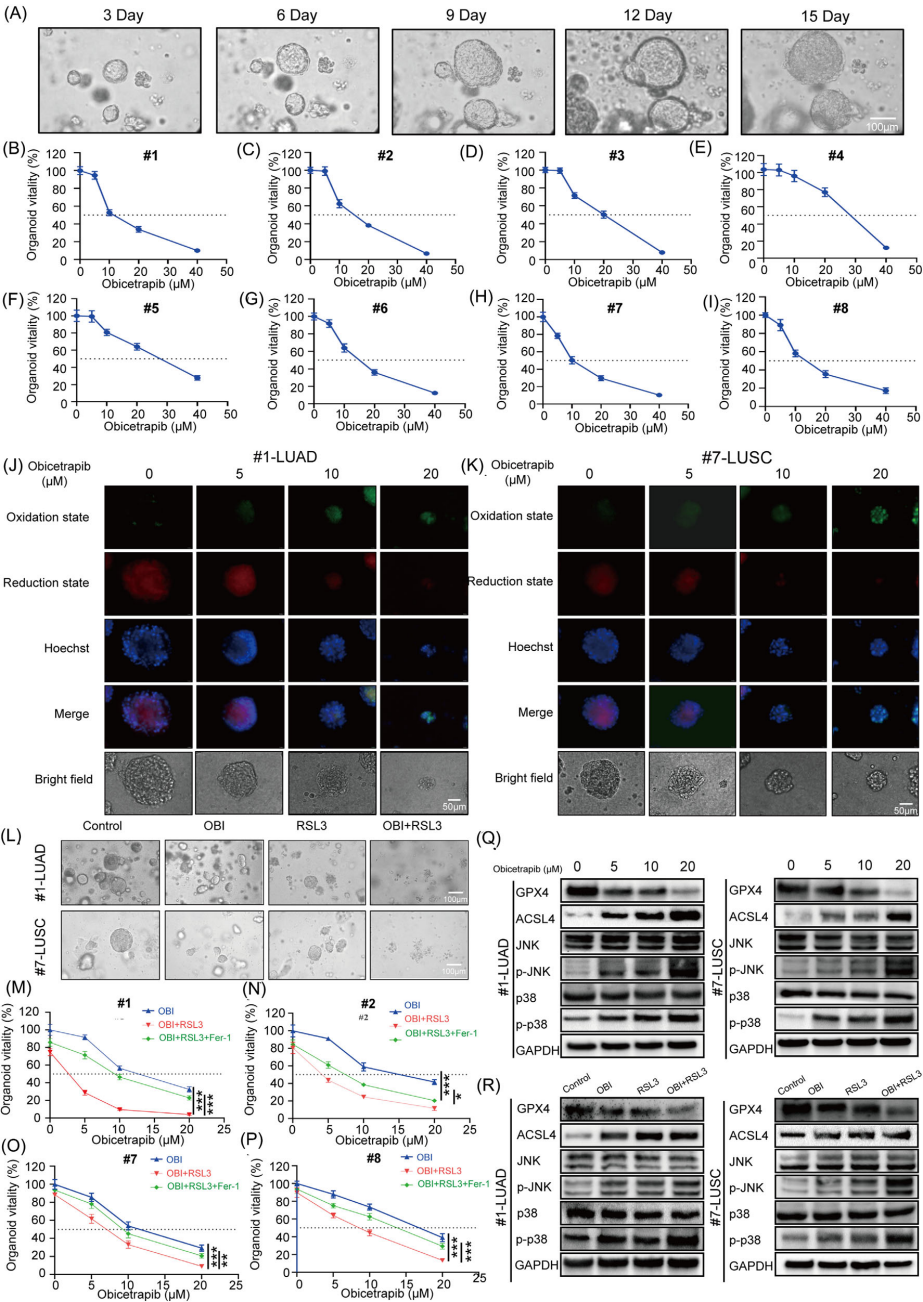
Patient‐derived organoid models for evaluating the ferroptosis‐regulating effects of obicetrapib in NSCLC. (A) Establishment of six lung adenocarcinoma (#1–#6) and two lung squamous cell carcinoma (#7–#8) organoid models. Scale bar, 100 µm. (B–I) Quantification of organoid size and viability following obicetrapib treatment. (J and K) Measurement of lipid peroxidation levels in lung adenocarcinoma and lung squamous cell carcinoma organoids treated with obicetrapib. Scale bar, 50 µm. (L) Bright‐field images of LUAD organoids treated with control, obicetrapib (10 µM), RSL3 (2.5 µM) or their combination. Scale bar, 100 µm. (M–P) Quantitative analysis of LUAD (#1, #2) and LUSC (#7, #8) organoid viability following treatment with dose‐escalating obicetrapib alone or in combination with 2.5 µM RSL3 or 2 µM ferrostatin‐1 (pre‐treated for 18 h). Organoid viability was assessed after 7 days of treatment. (Q and R) Western blot analysis of the expression of GPX4, ACSL4 and the phosphorylation of JNK and p38 in organoids of lung adenocarcinoma and lung squamous cell carcinoma with different concentration gradients of obicetrapib (Q) and combination therapy (R). Fer‐1, ferrostatin‐1; OBI, obicetrapib. All data represent mean ± SD from at least three independent experiments. *p < .05, **p < .01, ***p < .001, NS, not significant.

Building upon the in vitro and in vivo findings, we next utilised PDO models to validate the ferroptosis‐regulating effects of CETP inhibition in NSCLC. These models closely mimic the histological and biological features of primary tumours, offering a robust platform for functional verification. A total of six lung squamous cell carcinoma (#1–#6) and two lung squamous carcinoma (#7–#8) organoids were successfully established (Figure 8A). Immunohistochemical analysis confirmed that the organoids retained high histological similarity to their parental tumours (Figure S8A,B). Treatment with the CETP inhibitor obicetrapib markedly reduced organoid size and viability compared with the control group, indicating a strong inhibitory effect on tumour growth (Figure 8B–I). Obicetrapib treatment also led to significantly elevated lipid peroxidation levels, consistent with enhanced ferroptotic activity (Figure 8J,K). Furthermore, combined treatment with obicetrapib and the ferroptosis inducer, RSL3 produced the most pronounced inhibitory effects. In both major NSCLC histological subtypes, including lung adenocarcinoma and lung squamous cell carcinoma, PDOs in the combination group exhibited significantly smaller size and reduced viability compared with both the single‐agent and control groups (Figures 8L and S9A). Consistently, cell viability assays showed that combined treatment with obicetrapib and RSL3 markedly reduced cell survival, and this reduction was significantly rescued by Fer‐1 (Figures 8M–P and S9B–E). Western blot analysis of drug‐treated organoids revealed a dose‐dependent decrease in GPX4 expression, ACSL4 expression and and a corresponding increase in JNK/p38 phosphorylation with escalating concentrations of obicetrapib (Figures 8Q and S9F). Moreover, the combination of obicetrapib and RSL3 further enhanced GPX4 suppression and JNK/p38 activation compared with either agent alone (Figures 8R and S9G). These findings reinforce that CETP inhibition effectively sensitises NSCLC organoids to ferroptosis through suppression of GPX4, ACSL4 and activation of JNK/p38 signalling, thereby constraining tumour growth and viability.

## 4 DISCUSSION

This study identifies a previously unrecognised oncogenic pathway in NSCLC, in which the transcriptional co‐regulator CTBP1 rewiring lipid accumulation and suppressing ferroptosis thereby drives tumour progression by activating CETP. We delineate a coherent CTBP1–CETP–LD–MAPK regulatory axis that integrates metabolic adaptation with redox homeostasis, ultimately supporting malignant progress. Pharmacological blockade of CETP with obicetrapib phenocopied genetic inhibition and synergised with ferroptosis inducers to suppress NSCLC progression in both xenograft and organoid models, underscoring CETP as a promising metabolic vulnerability in lung cancer.

CETP has historically been studied in the context of cardiovascular disease, where it mediates CE exchange between HDL and LDL.^18^, ^19^, ^20^ This process enriches the cholesterol core of circulating LDL particles, facilitating their rapid internalisation into cells. Intracellularly, synthesised or recycled CETP functions as a lipid chaperone within endosomal networks. It directs endocytosed LDL‐cholesterol into cytoplasmic LDs while suppressing structural efflux and hydrolysis^58^, ^59^ Its oncogenic role, however, has remained obscure. Our findings reveal that CETP overexpression promotes intracellular LDL accumulation and LDs biogenesis—hallmarks of metabolic reprogramming that support tumour growth by fuelling membrane synthesis, energy production and redox buffering.^9^, ^60^, ^61^ Functionally, CETP suppresses ferroptosis by reducing intracellular Fe^2^^+^, ROS and lipid peroxidation, while enhancing GPX4 expression. These effects collectively mitigate lipid oxidative stress and protect tumour cells from ferroptotic death. The inverse regulation of these markers by CETP knockdown or pharmacologic inhibition establishes a causal link between CETP‐mediated lipid transport activity and ferroptosis resistance in NSCLC. LDL, a metabolite that is abundantly present both in circulation and within the tumour microenvironment, has recently been implicated in the regulation of tumour progression through modulation of immune responses.^62^, ^63^ Previous studies have demonstrated that lowering LDL‐C levels leads to a marked reduction in myeloid‐derived suppressor cells and M2‐polarised tumour‐associated macrophages within hepatic tumours, both of which represent major immunosuppressive populations in the tumour microenvironment. Conversely, aberrant LDL‐C metabolism facilitates tumour progression by modulating the differentiation and function of myeloid cells.^62^ Interestingly, Sun et al. demonstrated that LDL can interact with the T‐cell surface receptor LRP11, activating intracellular signalling pathways that enhance T‐cell proliferation and cytotoxicity while prolonging their effector function within tumours.^63^ However, as their study examined the entire LDL complex, it remains unclear whether these immunomodulatory effects arise from the cholesterol component or the associated apolipoproteins. Given that CETP is a key regulator of LDL metabolism, how it modulates and reshapes lipid metabolism and immune interactions within the broader tumour microenvironment remains unclear and warrants further investigation.

Although CTBP1 was initially characterised as a transcriptional co‐repressor, subsequent studies have shown that it can also function as a transcriptional activator under specific cellular or epigenetic contexts.^23^, ^32^, ^33^, ^34^ In the present study, we demonstrate that CTBP1 acts as a transcriptional activator, rather than a classical repressor, by binding to the promoter region of CETP. CTBP1 facilitates chromatin remodelling, promoting the enrichment of H3K4me3 at the CETP promoter and establishing a transcriptionally permissive chromatin state. To gain further mechanistic insight, we analysed CTBP1 protein interactions using STRING and GeneMANIA (Figure S10A,B). In addition to known transcriptional repressors such as histone deacetylase HDAC1 and HDAC2^64^ CTBP1 was predicted to interact with transcription factors traditionally associated with activation, including ZEB1 and ZNF217^46^, ^65^, ^66^, ^67^, ^68^

Taken together, these findings suggest that CTBP1 is not strictly a transcriptional repressor, but rather a context‐dependent regulator capable of bidirectional control of gene transcription. We speculate that this dual functionality may depend on specific biological contexts, such as cancer, inflammation or cellular stress. The regulatory relationship between CTBP1 and CETP revealed in our study highlights a novel mechanism by which CTBP1 can drive oncogenic transcription and links epigenetic regulation with lipid accumulation in NSCLC.

Our mechanistic analyses further demonstrate that CETP and CTBP1 converge on the JNK/p38 MAPK axis, a central redox‐sensitive signalling hub that modulates ferroptotic susceptibility. The inhibition of JNK and p38 phosphorylation by CTBP1 or CETP overexpression, together with enhanced activation upon CETP blockade or knockdown, suggests that CETP indirectly restrains ferroptosis by dampening MAPK‐driven oxidative signalling. Given that JNK and p38 activation are known to amplify lipid peroxidation and iron metabolism, our findings position CETP as a suppressor of ferroptotic signalling flux. Restoration of CETP expression in CTBP1‐depleted cells rescued MAPK suppression, GPX4 expression and oncogenic phenotypes, confirming the hierarchical control exerted by CTBP1 upstream of CETP in ferroptosis regulation.

While our findings firmly establish the functional intersection between the CTBP1–CETP axis and MAPK pathway modulation, we recognise that this regulatory cascade likely encompasses both direct metabolic effectors and indirect genetic networks. The vital contribution of lipid metabolism reprogramming driven by CETP is paramount; enhanced CETP‐mediated lipid accumulation substantially alters the cellular lipidome, thereby functionally suppressing the stress‐responsive branches of the MAPK pathway—specifically through the inhibition of p38 and JNK phosphorylation—to orchestrate robust ferroptosis resistance. Concurrently, as a multi‐target transcriptional co‐activator, CTBP1 potentially commands a broader downstream gene circuitry, modulating parallel signalling intermediates that indirectly feed into this cascade. Nonetheless, it is worth noting that the exhaustive upstream dissection of the complex MAPK signalling network lies beyond the primary scope of this investigation, which remains fundamentally focused on characterising the oncogenic functions of the CTBP1–CETP axis and validating its critical translational implications for clinical drug repurposing in NSCLC. Future targeted lipidomic investigations will be essential to fully delineate the precise biophysical interlocks of this metabolic–signalling crosstalk.

Preclinical studies identify CETP inhibition as a viable therapeutic strategy to re‐sensitise NSCLC cells to ferroptosis. Treatment with obicetrapib, a next‐generation CETP inhibitor currently in Phase III cardiovascular outcome trials.^21^, ^22^ effectively suppressed tumour growth and enhanced ferroptosis in vitro, in vivo and in PDO models. To better align with current clinical dosing practices, we performed in vivo experiments using a mouse NSCLC xenograft model with a clinically relevant dosing regimen. Obicetrapib is administered orally at 10 mg/day in ongoing cardiovascular clinical trials.^22^; accordingly, we adopted a daily oral gavage schedule in mice and calculated the dose based on BSA conversion, while also accounting for the higher metabolic rate in rodents. This treatment strategy resulted in a final dose of 5 µg/g (5 mg/kg), which significantly suppressed tumour growth in our xenograft models. Importantly, combination therapy with obicetrapib and the ferroptosis inducer RSL3 yielded synergistic anti‐tumour effects without inducing systemic toxicity, emphasising the translational feasibility of targeting lipid–ferroptosis crosstalk. A paramount advantage of exploiting the CETP inhibitor obicetrapib in this study lies in its compelling rationale for drug repurposing, which is firmly grounded in an established, rigorous clinical safety framework. While first‐generation CETP inhibitors were historically hampered by compound‐specific, off‐target toxicities—such as torcetrapib‐induced aldosterone activation and subsequent hypertension—or long‐term adipose accumulation due to extreme lipophilicity (such as anacetrapib), recent large‐scale Mendelian randomisation studies and target‐validation trials have demonstrated that these historical setbacks were molecule‐specific rather than drug‐target failures.^18^, ^19^ As a highly selective, next‐generation inhibitor, obicetrapib features optimised pharmacodynamics and hydrophilicity that eliminate these off‐target liabilities.^20^ Notably, recent landmark Phase III clinical trials, including the multinational BROADWAY cohort involving 2530 high‐risk patients.^22^ and the TANDEM trial.^21^ have validated that a standard human regimen of 10 mg once daily is exceptionally well tolerated, yielding adverse event, hepatic and renal safety profiles virtually indistinguishable from placebo.^21^, ^22^ Given its proven safety and strong lipid‐lowering efficacy in cardiovascular studies, along with the convenience of oral administration, obicetrapib represents a promising agent for translational application in oncology.^21^, ^22^

It is noteworthy that CETP high expression emerged as a critical prognostic indicator of poor survival across NSCLC patients. Correspondingly, tissue‐level characterisation revealed that CETP expression was significantly up‐regulated in NSCLC tumour tissues compared with adjacent normal counterparts, with its abundance positively correlating with advanced clinical stages. This clinicopathological coupling suggests a unified metabolic dependency wherein aggressive NSCLC cells overexpress CETP to accelerate the accumulation of LDL‐C. Consistently, in our PDO platforms encompassing LUAD (*n* = 6) and LUSC (*n* = 2), obicetrapib demonstrated potent growth‐inhibitory efficacy across both histopathological subtypes without evidence of subclass‐specific resistance. While these findings support the anti‐tumour potential of CETP inhibition and its ability to enhance ferroptosis‐based therapies, the precise therapeutic window and optimal dosing required for clinical efficacy in oncology remain to be established through future pharmacokinetic and clinical studies. These results not only extend the therapeutic potential of CETP inhibitors beyond cardiovascular disease but also highlight their role in overcoming metabolic plasticity and ferroptosis resistance in cancer.

From a clinical translation perspective, evaluating the safety boundary of CETP inhibitors in patients with advanced malignancies is essential, as these individuals frequently present with cancer cachexia, malnutrition or hypocholesterolemia. To date, no clinical trial data exist regarding the application of CETP inhibitors in patients with cancer cachexia; all milestone trials of this class—including the landmark REVEAL trial^18^ the Phase III BROADWAY trial^22^ the Phase III TANDEM trial^21^ and the Phase II ROSE trial^20^—have exclusively evaluated cardiovascular or dyslipidemic cohorts while explicitly excluding patients with active malignancies. Theoretically, however, the precise pharmacodynamics of CETP inhibitors suggest a minimal adverse impact on the basal lipid status of malnourished individuals. Unlike statins, CETP inhibitors do not impair cell‐intrinsic de novo cholesterol synthesis pathways; rather, they modulate intravascular neutral lipid exchange and accelerate the receptor‐mediated clearance of ApoB‐containing particles^18^, ^20^ Robust data from the BROADWAY and ROSE trials confirm that even when potent CETP inhibition by obicetrapib reduces circulating LDL‐C and ApoB to historic nadirs, the therapy maintains an acceptable safety profile without triggering hepatic, renal or systemic muscle toxicities^20^, ^22^ Because normal somatic cells can maintain baseline homeostasis through internal synthesis, CETP inhibition is theoretically safeguarded against systemic tissue exhaustion in low‐lipid environments. Nevertheless, whether actual cancer patients with severe cachexia experience distinct metabolic or systemic effects remains a key question for future clinical oncology studies.

In summary, this study reveals a novel metabolic–epigenetic regulatory network in NSCLC wherein CTBP1‐driven CETP activation orchestrates ferroptosis resistance through MAPK suppression and GPX4 up‐regulation. Pharmacologic CETP inhibition reinstates ferroptotic vulnerability and curtails tumour growth, providing a compelling translational rationale for repurposing CETP inhibitors such as obicetrapib in the treatment of lipid‐dependent, ferroptosis‐resistant lung cancer.

## AUTHOR CONTRIBUTIONS

Yanjie Chen, Heng Wang and Ximin Tan performed the experiments. Chenxi Yan collected patient samples. Fangfang Liu, Shuxuan Deng and Yangcheng Xia performed bioinformatics data analysis. Zhaolin Xu, Chengyan Wang and Chenxi Yan assisted the experiments and provided reagents. Qian Chu, Shanshan Huang and Kongming Wu supervised the study. Qian Chu, Shanshan Huang and Yanjie Chen designed the study, wrote and revised the manuscript with inputs from other co‐authors. All authors read and approved the final manuscript.

## CONFLICT OF INTEREST STATEMENT

The authors declare no conflicts of interest.

## FUNDING INFORMATION

The authors have nothing to report.

## Supporting information



Supporting Information

Supporting Information

## Data Availability

All the data supporting this article are available within the article and its supplementary materials. The raw data will also be made available on request.
