# Supplementary material for: Targeting the CTBP1–CETP axis overcomes ferroptosis resistance in non‐small cell lung cancer by altering lipid accumulation
Source: Clin Transl Med. 2026 Jul 27;16(8):e70749. doi: 10.1002/ctm2.70749 (PMC13408231; doi:10.1002/ctm2.70749)
Supplement: Supplementary file 1 — Supporting Information [file CTM2-16-e70749-s001.docx]

**Supplementary Figure**

**
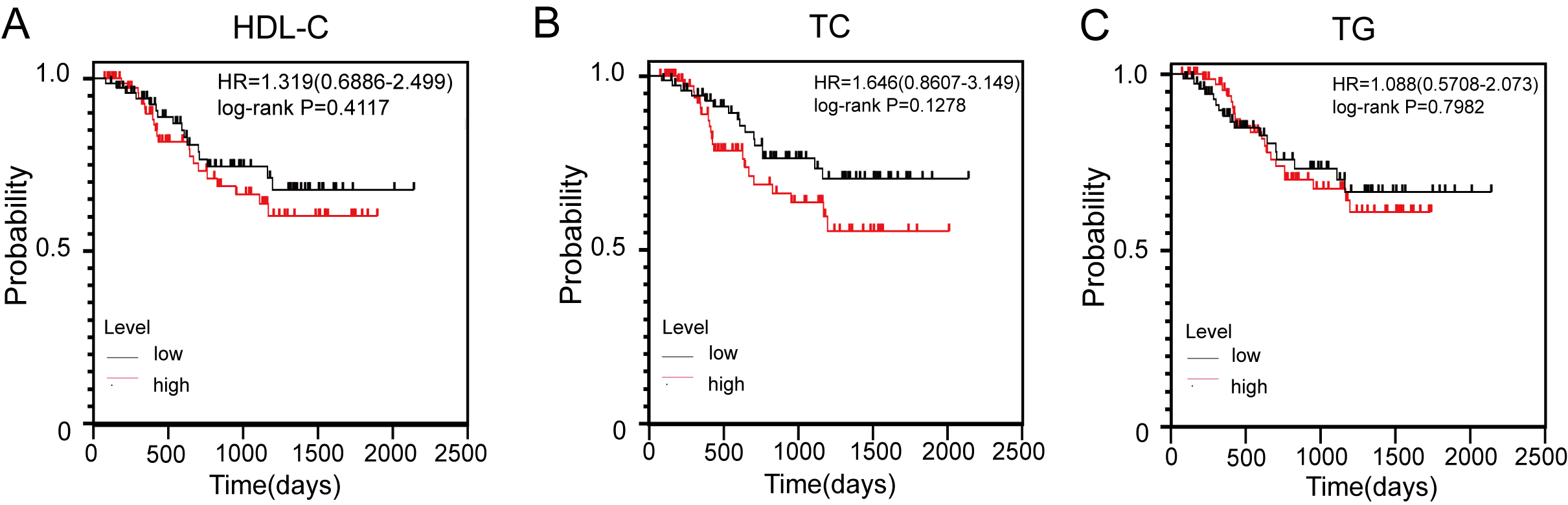
**

**Supplementary Figure 1. Association of clinical lipid levels with overall survival in NSCLC patients.
(A)** Kaplan–Meier survival curves for OS of 151 NSCLC patients stratified by HDL-C levels. **(B)** Kaplan–Meier survival curves for OS of 151 NSCLC patients stratified by TC levels. **(C)** Kaplan–Meier survival curves for OS of 151 NSCLC patients stratified by TG levels.


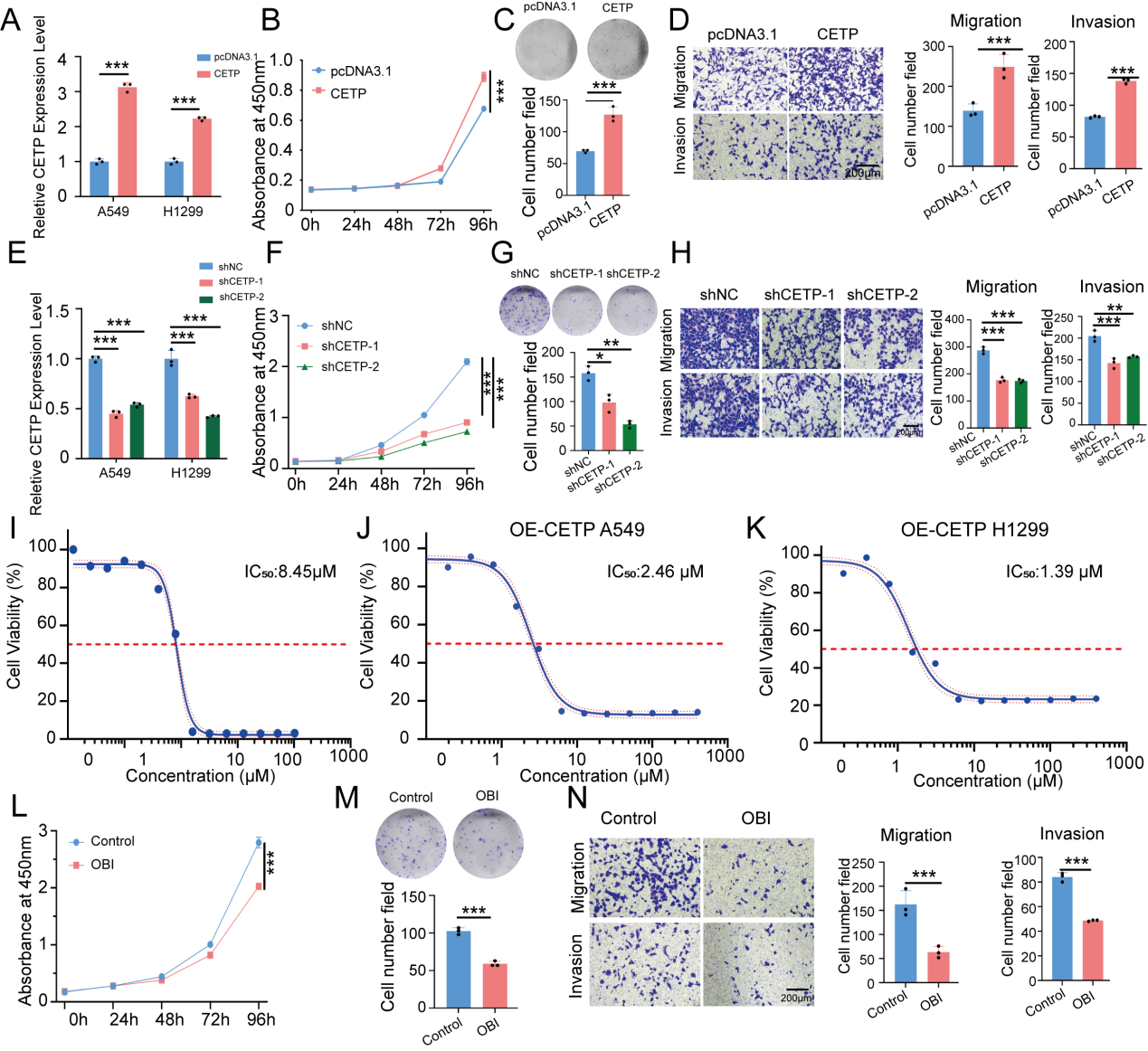


**Supplementary Figure 2. CETP exerts a tumor-promoting role in H1299 cells.**

1. Densitometric analysis of Western blot band intensities in A549 and H1299 cells following overexpression CETP. Protein expression levels were normalized to GAPDH. **(B)** CCK-8 assays showed the growth of H1299 cells upon CETP overexpression. **(C)** The effect of CETP overexpression on the colony formation of H1299 cells. **(D)** Transwell cell migration and invasion analysis of CETP overexpressed and control H1299 cells. Scale bar, 200 μm. **(E)** Western blot analysis of the indicated proteins in knockdown CETP A549 and H1299 cells. Protein expression levels were normalized to GAPDH.**(F)** The effect of CETP knockdown on H1299 cell growth was determined by CCK-8 assays. **(G)** Colony formation assays were performed in CETP knockdown and control cells. **(H)** Migration and invasion assays of H1299 cells with or without CETP knockdown. Scale bar, 200 μm. **(I)** Determination of the IC_50_ of Obicetrapib in H1299 cells. **(J-K)** Determination of the IC_50_ of Obicetrapib in CETP overexpression A549 and H1299 cells. **(L-M)** Cell viability and colony formation assays in H1299 cells treated with Obicetrapib at 1/2 IC_50_. **(N)** Transwell migration and invasion assays of H1299 cells under the 1/2 IC_50_ Obicetrapib treatment conditions. Scale bar, 200 μm. All data represent mean ± SD from at least three independent experiments. **P < .01, ***P < .001, NS, not significant.


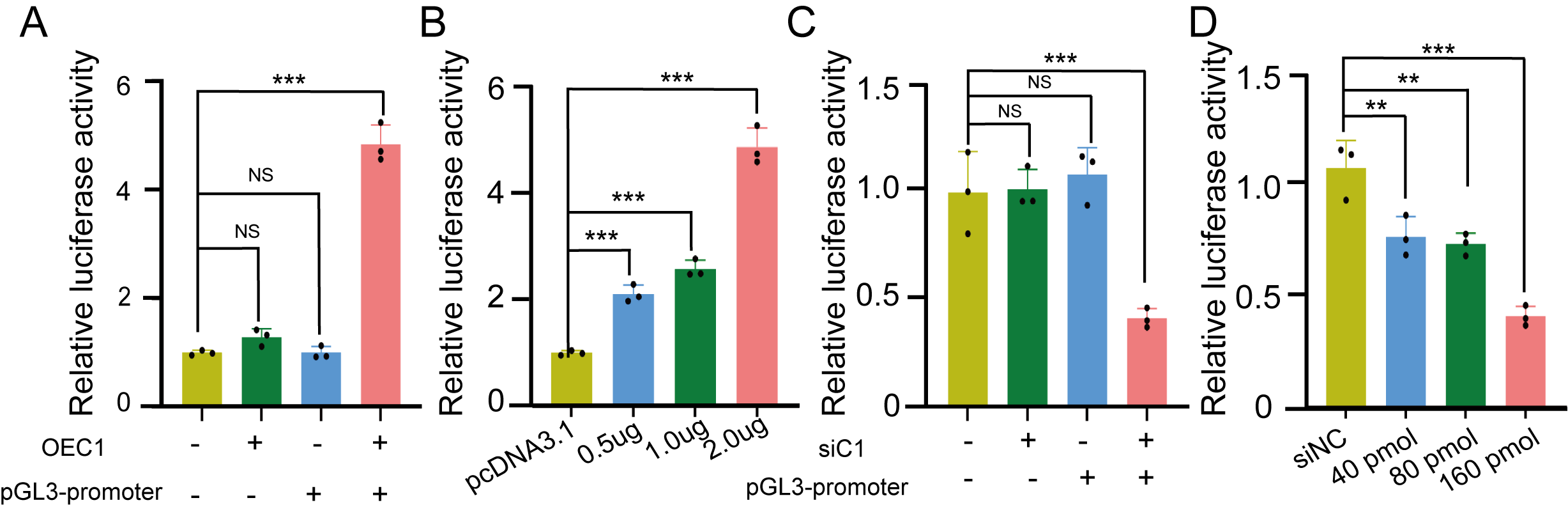


**Supplementary Figure 3. CTBP1 activates CETP transcription in H1299 cells.**

**(A-B)** Luciferase reporter assays showing a dose-dependent increase in CETP promoter activity upon CTBP1 overexpression. **(C-D)** Luciferase reporter assays showing a graded decrease in promoter activity after siRNA-mediated CTBP1 knockdown. All data represent mean ± SD from at least three independent experiments. *P < .05, **P < .01, ***P < .001, NS, not significant.


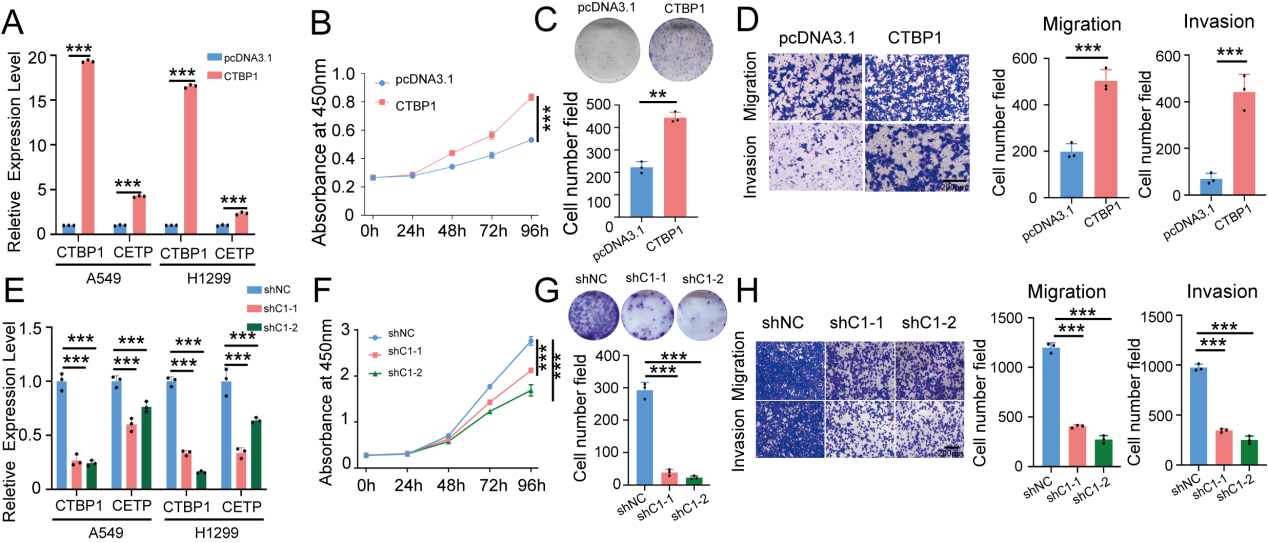


**Supplementary Figure 4. CTBP1 facilitates H1299 cells malignant progression.**

1. Western blot analysis of CTBP1 and CETP expression in CTBP1 overexpression NSCLC cells. Protein expression levels were normalized to GAPDH. **(B)** Cell growth curves of H1299 cells with or without CTBP1 overexpression by CCK-8 assays. **(C)** CTBP1 contributes to the proliferation of H1299 cells as measured by colony formation assays. **(D)** Transwell cell migration and invasion analysis of CTBP1 overexpressed and control H1299 cells. Scale bar, 200 μm. **(E)** Western blot analysis of CTBP1and CETP expression in CTBP1 knockdown A549 and H1299 cells. Protein expression levels were normalized to GAPDH. **(F)** The effect of CTBP1 knockdown on H1299 cell growth was determined by CCK-8 assays. **(G)** Colony formation assays were performed in CTBP1 knockdown and control cells. **(H)** Migration and invasion assays of H1299 cells with or without CTBP1 knockdown. Scale bar, 200 μm. Abbreviations: shC1; shCTBP1. All data represent mean ± SD from at least three independent experiments. *P < .05, **P < .01, ***P < .001, NS, not significant.


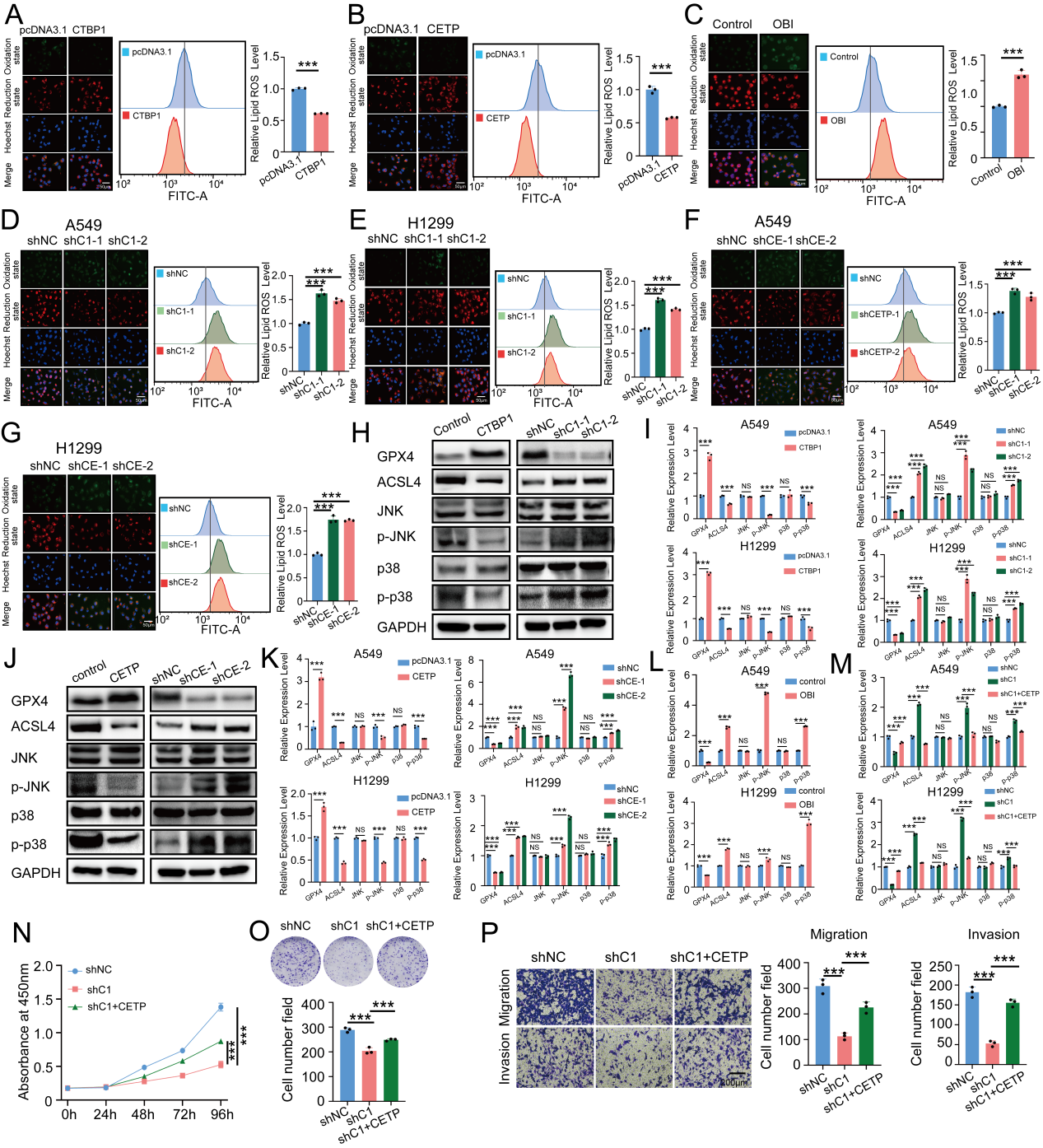


**Supplementary Figure 5. Experimental analysis of the CTBP1–CETP axis in regulating ferroptosis and MAPK signaling in NSCLC cells.**

**(A–B)** Intracellular lipid peroxidation levels detected by BODIPY 581/591 C11 assay staining and flow cytometry in CTBP1 and CETP overexpression H1299 cells. Scale bar, 50 μm. **(C)** Intracellular lipid peroxidation levels in H1299 cells treated with Obicetrapib at 1/2 the IC_50_ concentration were evaluated by BODIPY 581/591 C11 Assay staining and quantified by flow cytometry. Scale bar, 50 μm. **(D-E)** BODIPY 581/591 C11 Assay staining combined with flow cytometry was used to evaluate intracellular lipid peroxidation levels of CTBP1 deletion A549 and H1299 cells. Scale bar, 50 μm. **(F-G)** BODIPY 581/591 C11 Assay staining combined with flow cytometry was used to evaluate intracellular lipid peroxidation levels of CETP deletion A549 and H1299 cells. Scale bar, 50 μm. **(H)** Western blot analysis of GPX4/ACSL44 expression and JNK/p38 phosphorylation in response to CTBP1 modulation in H1299 cells. **(I)** Densitometric quantification of protein expression levels in (H). Protein expression levels were normalized to GAPDH. **(J)** Western blot analysis of GPX4, ACSL4, and JNK/p38 phosphorylation in H1299 cells with CETP overexpression or knockdown. **(K)** Densitometric quantification of (J) figure protein expression levels. Protein expression levels were normalized to GAPDH. **(L)** Densitometric analysis of Western blot band intensities in A549 and H1299 cells following treatment with 1/2 IC_50_ Obicetrapib. Protein expression levels were normalized to GAPDH. **(M)** Quantification of protein expression levels in A549 and H1299 cells under CTBP1 knockdown and rescue conditions. Protein expression levels were normalized to GAPDH. **(N)** Overexpression of CETP rescues the cell proliferation suppressed by CTBP1 knockdown in H1299 cells by CCK-8 assays. **(O)** Overexpression of CETP restores the colony formation of H1299 cells suppressed by CTBP1 knockdown. **(P)** Cell migration and invasion analysis of CTBP1-depleted H1299 cells with or without CETP re-expression. Scale bar, 200 μm. Abbreviations: shC1, shCTBP1; shCE, shCETP. All data represent mean ± SD from at least three independent experiments. *P < .05, **P < .01, ***P < .001, NS, not significant.


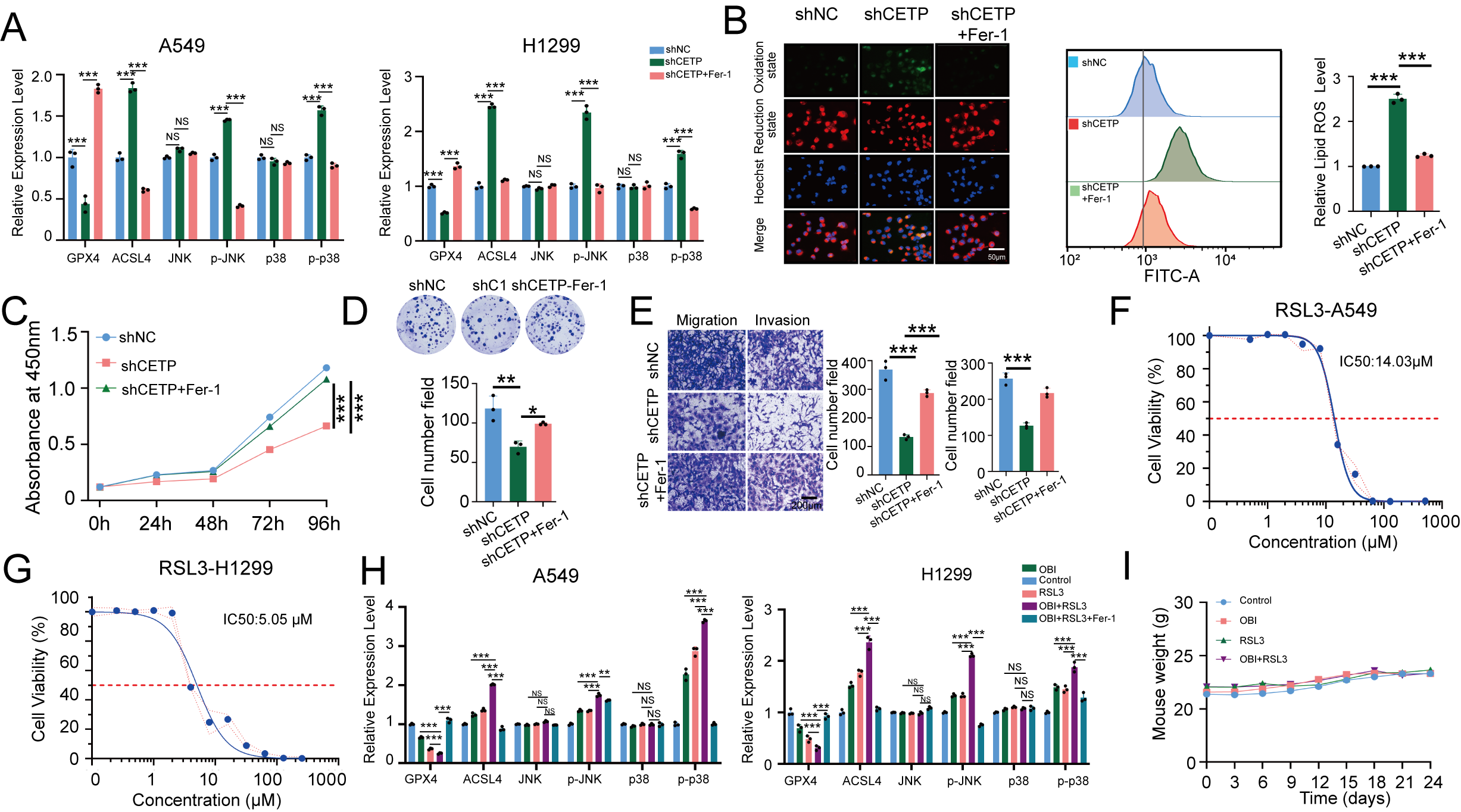


**Supplementary Figure 6. CETP regulates ferroptosis and modulates the therapeutic response to Obicetrapib in NSCLC.**

1. Quantification of protein expression levels in A549 and H1299 cells under CETP knockdown following treatment with the ferroptosis inhibitor Fer-1. Protein expression levels were normalized to GAPDH. **(B)** Lipid peroxidation detected by BODIPY 581/591 C11 staining in CETP-knockdown H1299 cells following Fer-1 treatment. **(C-D)** Rescue of cell viability and colony formation by Fer-1 in CETP-knockdown H1299 cells. **(E)** Transwell migration and invasion assays in CETP-knockdown H1299 cells following Fer-1 treatment. Scale bar, 200 μm. **(F-G)** IC_50_ determination for RSL3 in A549 and H1299 cells. **(H)** Quantification of Western blot band intensities in A549 and H1299 cells treated with Control, OBI, RSL3, OBI+RSL3, or OBI+RSL3+Fer-1. Protein expression levels were normalized to GAPDH. **(I)** Body weight changes of mice treated with OBI, RSL3, OBI+RSL3, or vehicle control. Abbreviations: OBI, obicetrapib; Fer-1, ferrostatin-1. All data represent mean ± SD from at least three independent experiments. ***P < .001, NS, not significant.

**
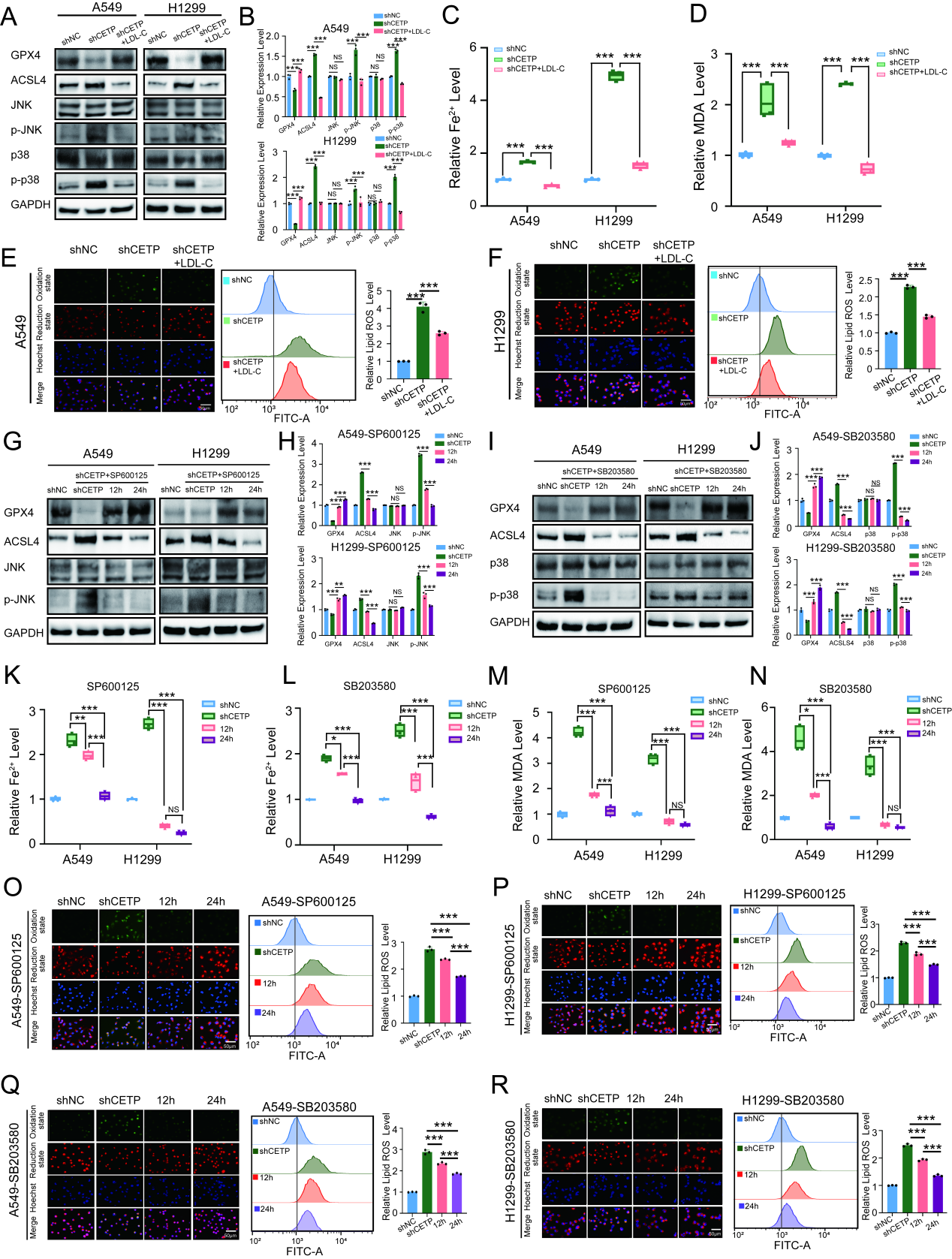
**

**Supplementary Figure 7. CETP knockdown promotes ferroptosis via lipid deprivation and JNK/p38 MAPK pathway activation.**

1. Protein expression in A549 and H1299 cells under CETP knockdown following treatment with the cholesteryl linoleate. **(B)** Quantification of (A) figure Western blot band intensities. Protein expression levels were normalized to GAPDH. **(C)** Quantification of intracellular Fe²+ levels in NSCLC cells following treatment with the cholesteryl linoleate. **(D)**  Quantification of intracellular MDA levels in A549 and H1299 cells following treatment with the cholesteryl linoleate. **(E-F)** Quantification and visualization of intracellular lipid peroxidation in A549 and H1299 cells using the C11-BODIPY probe under CETP knockdown or exogenous cholesteryl linoleate treatment. Scale bar, 50 μm. **(G-H)** Western blot analysis (G) and corresponding densitometric quantification (H) of the indicated proteins in A549 and H1299 cells following treatment with the JNK inhibitor (SP600125) for 12 and 24 hours under CETP knockdown conditions. Protein expression levels were normalized to GAPDH. **(I-J)** Western blot analysis (I) and corresponding densitometric quantification (J) of the indicated proteins in A549 and H1299 cells following treatment with the p38 inhibitor (SB203580) for 12 and 24 hours under CETP knockdown conditions. Protein expression levels were normalized to GAPDH. **(K-L)** Quantification of intracellular Fe^2+^ levels in NSCLC cells following treatment with SP600125 or SB203580. **(M-N)** Quantification of intracellular MDA levels in NSCLC cells following treatment with SP600125 or SB203580. **(O-P)** C11-BODIPY staining and flow cytometry analysis of intracellular lipid peroxidation in A549 and H1299 cells treated with SP600125 (12 and 24 h) under CETP knockdown conditions. Scale bar, 50 μm. **(Q-R)** Intracellular lipid peroxidation levels in A549 and H1299 cells under CETP knockdown and SB203580 treatment (12 and 24 h), assessed by C11-BODIPY staining and flow cytometry. Scale bar, 50 μm. Abbreviations: cholesteryl linoleate, LDL-C; 12h, 12 hours, 24h, 24 hours. All data represent mean ± SD from at least three independent experiments.*< .05, **P < .01, ***P < .001, NS, not significant.


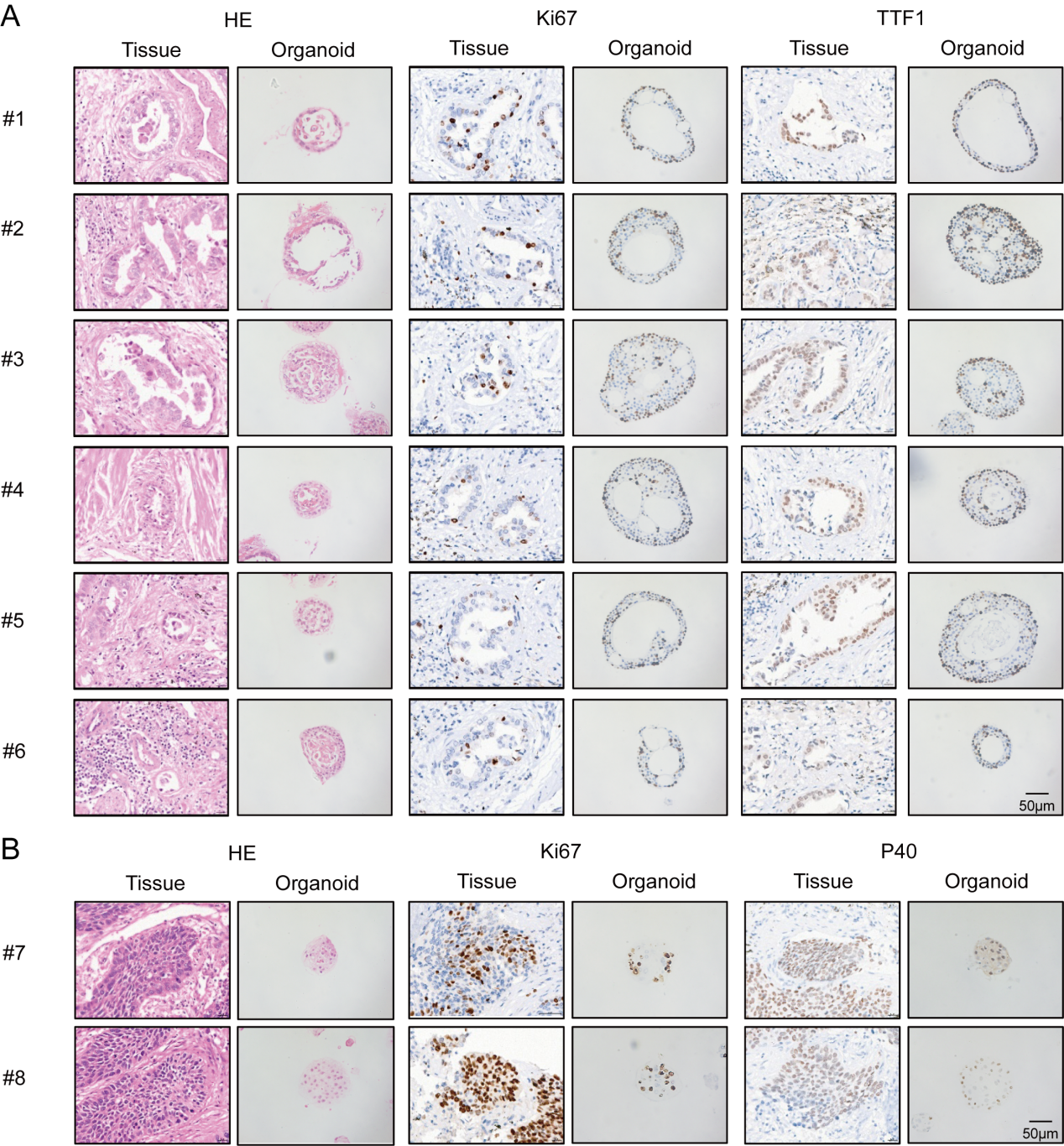


**Supplementary Figure 8. Pathological features of NSCLC organoids compared with those of the original patient tumors tissue.**

1. Immunohistochemical analysis of 6 lung adenocarcinoma organoids and their matched parental tumor tissues. Scale bar, 50 μm. **(B)** Immunohistochemical analysis of 2 lung squamous carcinoma organoids and their matched parental tumor tissues. Scale bar, 50 μm.

**
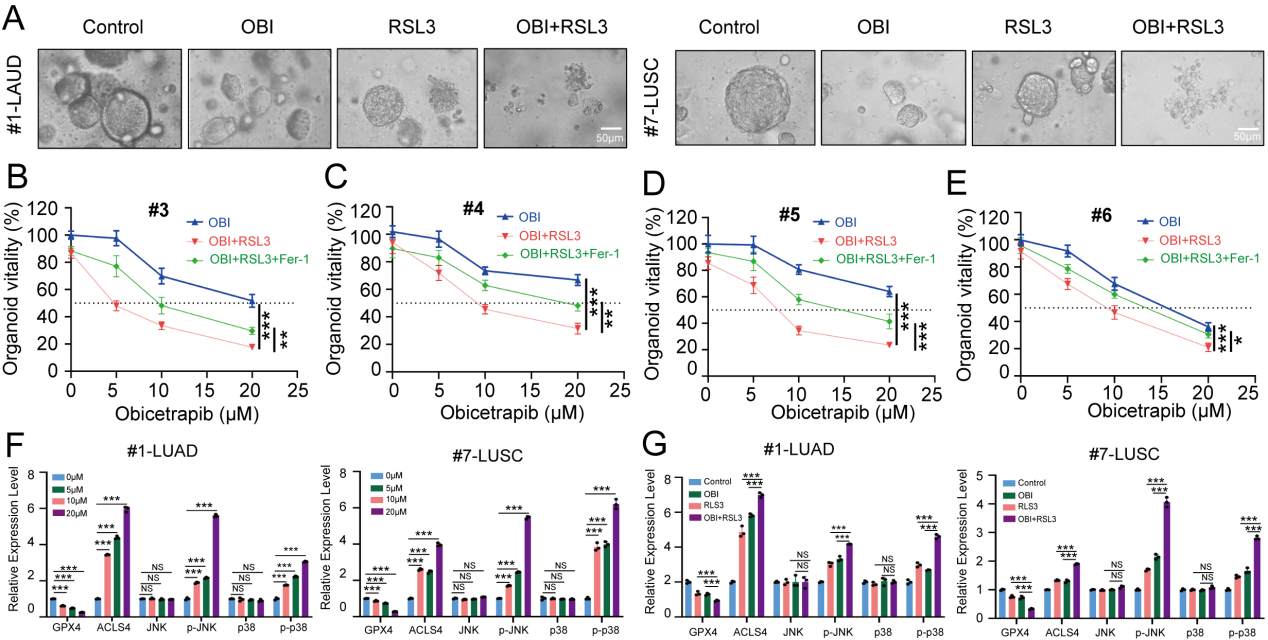
**

**Supplementary Figure 9. Obicetrapib sensitizes NSCLC patient-derived organoids to ferroptosis**

**(A)** Bright-field images of LUAD and LUSC organoids treated with Obicetrapib (10 μM) alone or in combination with RSL3 (2.5μM). Scale bar, 50 μm. **(B-E)** Quantitative analysis of #3-#6 organoid viability following treatment with dose-escalating Obicetrapib alone, or in combination with 2.5 μM RSL3 or 2 μM ferrostatin-1 (pre-treated for 18 hours). Organoid viability was assessed after 7 days of treatment. **(F)** Protein expression in #1-LUAD and #7-LUSC organoids treated with SP600125 (12 or 24 hours) under CETP knockdown conditions. Protein expression levels were normalized to GAPDH. **(G)** Protein expression in #1-LUAD and #7-LUSC organoids under CETP knockdown following SB203580 treatment for 12 or 24 hours. Protein expression levels were normalized to GAPDH. Abbreviations: OBI, obicetrapib; Fer-1, ferrostatin-1. All data represent mean ± SD from at least three independent experiments. **P < .01, ***P < .001, NS, not significant.


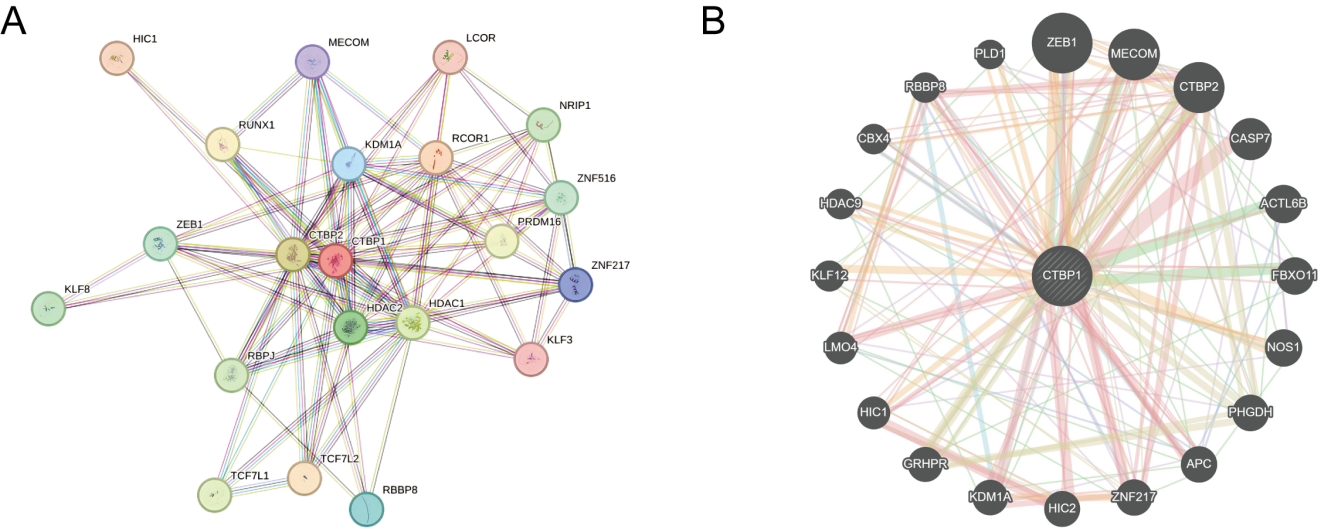


**Supplementary Figure 10. Predicted CTBP1 interacting proteins.**

**(A)** Protein-protein interaction network of CTBP1 predicted using the STRING database. **(B)** CTBP1 interacting protein network predicted using the GeneMANIA online tool.
