# Supplementary material for: Targeting the CTBP1–CETP axis overcomes ferroptosis resistance in non‐small cell lung cancer by altering lipid accumulation
Source: Clin Transl Med. 2026 Jul 27;16(8):e70749. doi: 10.1002/ctm2.70749 (PMC13408231; doi:10.1002/ctm2.70749)
Supplement: Supplementary file 2 — Supporting Information [file CTM2-16-e70749-s002.docx]

| NSCLC tumor tissues | | | | | |
| --- | --- | --- | --- | --- | --- |
| Sample ID | TNM | Stages | Sample ID | TNM | Stages |
| LCA_1 | T3N2M0 | IIIB | LCA_28 | T2aN0M0 | IB |
| LCA_2 | T1cN0M0 | IA | LCA_29 | T2aN0M0 | IB |
| LCA_3 | T1bN0M0 | IA | LCA_30 | T2aN0M0 | IB |
| LCA_4 | T1bN0M0 | IA | LCA_31 | T2aN0M0 | IB |
| LCA_5 | T1bN0M0 | IA | LCA_32 | T2aN0M0 | IB |
| LCA_6 | T1cN0M0 | IA | LCA_33 | T2aN0M0 | IB |
| LCA_7 | T1bN0M0 | IA | LCA_34 | T2bN0M0 | IIA |
| LCA_8 | T1bN0M0 | IA | LCA_35 | T1cN1M0 | IIA |
| LCA_9 | T1cN0M0 | IA | LCA_36 | T3N0M0 | IIB |
| LCA_10 | T1bN0M0 | IA | LCA_37 | T3N0M0 | IIB |
| LCA_11 | T1bN0M0 | IA | LCA_38 | T3N0M0 | IIB |
| LCA_12 | T1aN0M0 | IA | LCA_39 | T3N0M0 | IIB |
| LCA_13 | T1bN0M0 | IA | LCA_40 | T2aN1M0 | IIB |
| LCA_14 | T1bN0M0 | IA | LCA_41 | T3N0M0 | IIB |
| LCA_15 | T1cN0M0 | IA | LCA_42 | T3N0M0 | IIB |
| LCA_16 | T1cN0M0 | IA | LCA_43 | T2aN2aM0 | IIIA |
| LCA_17 | T1bN0M0 | IA | LCA_44 | T2aN2aM0 | IIIA |
| LCA_18 | T1aN0M0 | IA | LCA_45 | T2bN2aM0 | IIIA |
| LCA_19 | T1bN0M0 | IA | LCA_46 | T2aN2aM0 | IIIA |
| LCA_20 | T1cN0M0 | IA | LCA_47 | T2aN2aM0 | IIIA |
| LCA_21 | T1aN0M0 | IA | LCA_48 | T1bN2bM0 | IIIA |
| LCA_22 | T1aN0M0 | IA | LCA_49 | T1bNb2M0 | IIIA |
| LCA_23 | T2aN0M0 | IB | LCA_50 | T1cN2bM0 | IIIA |
| LCA_24 | pT2aN0M0 | IB | LCA_51 | T3N2bM0 | IIIB |
| LCA_25 | T2aN0M0 | IB | LCA_52 | T4N2M0 | IIIB |
| LCA_26 | T2aN0M0 | IB | LCA_53 | T2N1M1a | IVA |
| LCA_27 | T2aN0M0 | IB | LCA_54 | T4N0M1a | IVA |

**Supplementary Table 1：**

**Clinical and pathological features of tumor tissues from NSCLC patients for RNA extraction**

| Normal lung tissues | | | |
| --- | --- | --- | --- |
| Sample ID | Pathological type | Sample ID | Pathological type |
| LN_1 | Normal | LN_25 | Normal |
| LN_2 | Normal | LN_26 | Normal |
| LN_3 | Normal | LN_27 | Normal |
| LN_4 | Normal | LN_28 | Normal |
| LN_5 | Normal | LN_29 | Normal |
| LN_6 | Normal | LN_30 | Normal |
| LN_7 | Normal | LN_31 | Normal |
| LN_8 | Normal | LN_32 | Normal |
| LN_9 | Normal | LN_33 | Normal |
| LN_10 | Normal | LN_34 | Normal |
| LN_11 | Normal | LN_35 | Normal |
| LN_12 | Normal | LN_36 | Normal |
| LN_13 | Normal | LN_37 | Normal |
| LN_14 | Normal | LN_38 | Normal |
| LN_15 | Normal | LN_39 | Normal |
| LN_16 | Normal | LN_40 | Normal |
| LN_17 | Normal | LN_41 | Normal |
| LN_18 | Normal | LN_42 | Normal |
| LN_19 | Normal | LN_43 | Normal |
| LN_20 | Normal | LN_44 | Normal |
| LN_21 | Normal | LN_45 | Normal |
| LN_22 | Normal | LN_46 | Normal |
| LN_23 | Normal | LN_47 | Normal |
| LN_24 | Normal |  |  |

| Sample ID | TNM | Stages | Sample ID | TNM | Stages |
| --- | --- | --- | --- | --- | --- |
| PLCA-1 | T2N1M0 | IIB | PLCA-29 | T2N1M0 | IIB |
| PLCA-2 | T3N2bM0 | IIIB | PLCA-30 | T3N2bM0 | IIIB |
| PLCA-3 | T3N0M0 | IIb | PLCA-31 | T3N0M0 | IIb |
| PLCA-4 | T2aN0M0 | IB | PLCA-32 | T2aN0M0 | IB |
| PLCA-5 | T3N0M0 | IIB | PLCA-33 | T3N0M0 | IIB |
| PLCA-6 | T3N1M0 | IIIA | PLCA-34 | T3N1M0 | IIIA |
| PLCA-7 | T3N1M0 | IIIA | PLCA-35 | T3N1M0 | IIIA |
| PLCA-8 | T4N1M0 | IIIA | PLCA-36 | T4N1M0 | IIIB |
| PLCA-9 | T2bN0M0 | IIA | PLCA-37 | T2bN0M0 | IIA |
| PLCA-10 | T4N0M1 | IV | PLCA-38 | T4N0M1 | IV |
| PLCA-11 | T4N2M0 | IIIB | PLCA-39 | T4N2M0 | IIIB |
| PLCA-12 | T2N1M0 | IIB | PLCA-40 | T2N1M0 | IIB |
| PLCA-13 | T2N2M0 | IIIA | PLCA-41 | T2N2aM0 | IIIA |
| PLCA-14 | T4N2M0 | IIIB | PLCA-42 | T4N2M0 | IIIB |
| PLCA-15 | T4N0M0 | IIIA | PLCA-43 | T4N0M0 | IIIA |
| PLCA-16 | T4N2M0 | IIIB | PLCA-44 | T4N2M0 | IIIB |
| PLCA-17 | T2bN0M0 | IIA | PLCA-45 | T2bN0M0 | IIA |
| PLCA-18 | T3N2bM0 | IIIB | PLCA-46 | T3N2bM0 | IIIB |
| PLCA-19 | T2aN0M0 | IB | PLCA-47 | T2aN0M0 | IB |
| PLCA-20 | T2bN2aM0 | IIIA | PLCA-48 | T2bN2M0 | IIIA |
| PLCA-21 | T2aN0M0 | IB | PLCA-49 | T2aN0M0 | IB |
| PLCA-22 | T2bN0M0 | IIA | PLCA-50 | T2bN0M0 | IIA |
| PLCA-23 | T1N0M0 | IA | PLCA-51 | T1N0M0 | IA |
| PLCA-24 | T4N0M0 | IIIA | PLCA-52 | T4N0M0 | IIIA |
| PLCA-25 | T2bN0M0 | IIA | PLCA-53 | T2bN0M0 | IIA |
| PLCA-26 | T4N2M0 | IIIB | PLCA-54 | T4N2M0 | IIIB |
| PLCA-27 | T4N1M0 | IIIA | PLCA-55 | T4N1M0 | IIIA |
| PLCA-28 | T4N2M0 | IIIB | PLCA-56 | T4N2M0 | IIIB |

**Supplementary Table 2：**

**Clinical and pathological features of tumor tissues from NSCLC patients for tissue microarrays**

| Sample ID | TNM | Stages | Sample ID | TNM | Stages |
| --- | --- | --- | --- | --- | --- |
| PLCA-57 | T4N0M0 | IIIA | PLCA-86 | T2N1M0 | IIB |
| PLCA-58 | T2bN0M0 | IIA | PLCA-87 | T4N1M0 | IIIA |
| PLCA-59 | T4N0M0 | IIIA | PLCA-88 | T2bN0M0 | IIA |
| PLCA-60 | T4N0M0 | IIIA | PLCA-89 | T2aN0M0 | IB |
| PLCA-61 | T1cN0M0 | IA | PLCA-90 | T4N0M0 | IIIA |
| PLCA-62 | T3N1M0 | IIIA | PLCA-91 | T4N2M0 | IIIB |
| PLCA-63 | T3N2bM0 | IIIB | PLCA-92 | T4N0M0 | IIIA |
| PLCA-64 | T4N2M0 | IIIB | PLCA-93 | T3N1M0 | IIIA |
| PLCA-65 | T1cN0M0 | IA | PLCA-94 | T4N0M0 | IIIA |
| PLCA-66 | T4N2M0 | IIIB | PLCA-95 | T3N2bM0 | IIIB |
| PLCA-67 | T4N2M1 | IV | PLCA-96 | T2N1M0 | IIB |
| PLCA-68 | T4N0M0 | IIIA | PLCA-97 | T4N0M0 | IIIA |
| PLCA-69 | T2aN0M0 | IB | PLCA-98 | T3N0M0 | IIB |
| PLCA-70 | T2aN0M0 | IB | PLCA-99 | T2aN0M0 | IB |
| PLCA-71 | T4N0M0 | IIIA | PLCA-100 | T4N2M0 | IIIB |
| PLCA-72 | T2aN0M0 | IB | PLCA-101 | T1N1M0 | IIB |
| PLCA-73 | T2aN2aM0 | IIIA | PLCA-102 | T3N1M0 | IIIA |
| PLCA-74 | T2aN0M0 | IB | PLCA-103 | T2bN0M0 | IIA |
| PLCA-75 | T3N1M0 | IIIA | PLCA-104 | T2aN2aM0 | IIIA |
| PLCA-76 | T4N1M0 | IIIA | PLCA-105 | T2aN0M0 | IB |
| PLCA-77 | T2bN0M0 | IIA | PLCA-106 | T3N0M0 | IIB |
| PLCA-78 | T2aN0M0 | IB | PLCA-107 | T2bN2aM0 | IIIA |
| PLCA-79 | T4N0M0 | IIIA | PLCA-108 | T3N0M0 | IIB |
| PLCA-80 | T2bN0M0 | IIA | PLCA-109 | T4N0M0 | IIIA |
| PLCA-81 | T3N1M0 | IIIA | PLCA-110 | T4N2M0 | IIIB |
| PLCA-82 | T3N1M0 | IIIA | PLCA-111 | T4N2M0 | IIIB |
| PLCA-83 | T2aN0M0 | IB | PLCA-112 | T3N2bM0 | IIIB |
| PLCA-84 | T2bN0M0 | IIA | PLCA-113 | T4N0M0 | IIIA |
| PLCA-85 | T4N2M0 | IIIB |  |  |  |

**Supplementary Table 3：**

| Sample ID | Stages | OS (days) | Survival state | Sample ID | Stages | OS (days) | Survival state |
| --- | --- | --- | --- | --- | --- | --- | --- |
| PL-1 | IV | 74 | 0 | PL-27 | IV | 298 | 1 |
| PL-2 | IIIB | 82 | 1 | PL-28 | IV | 301 | 1 |
| PL-3 | IV | 95 | 0 | PL-29 | IV | 321 | 1 |
| PL-4 | IV | 108 | 0 | PL-30 | IV | 325 | 0 |
| PL-5 | IV | 118 | 0 | PL-31 | IV | 332 | 0 |
| PL-6 | IV | 126 | 0 | PL-32 | IIIB | 337 | 0 |
| PL-7 | IV | 141 | 0 | PL-33 | IV | 344 | 1 |
| PL-8 | IV | 148 | 0 | PL-34 | IV | 348 | 1 |
| PL-9 | IV | 155 | 1 | PL-35 | IV | 349 | 0 |
| PL-10 | IV | 155 | 0 | PL-36 | IV | 356 | 0 |
| PL-11 | IV | 172 | 0 | PL-37 | IV | 357 | 0 |
| PL-12 | IV | 174 | 0 | PL-38 | IV | 376 | 1 |
| PL-13 | IIIC | 174 | 0 | PL-39 | IV | 376 | 0 |
| PL-14 | IV | 188 | 1 | PL-40 | IIIB | 377 | 0 |
| PL-15 | IV | 198 | 0 | PL-41 | IV | 384 | 0 |
| PL-16 | IIIB | 213 | 1 | PL-42 | IV | 386 | 0 |
| PL-17 | IV | 217 | 0 | PL-43 | IV | 393 | 0 |
| PL-18 | IV | 221 | 0 | PL-44 | IIIB | 395 | 1 |
| PL-19 | IV | 221 | 0 | PL-45 | IV | 403 | 1 |
| PL-20 | IV | 225 | 1 | PL-46 | IV | 411 | 0 |
| PL-21 | IV | 233 | 0 | PL-47 | IV | 413 | 1 |
| PL-22 | IV | 237 | 0 | PL-48 | IIIB | 418 | 1 |
| PL-23 | IV | 253 | 0 | PL-49 | IV | 421 | 0 |
| PL-24 | IV | 254 | 0 | PL-50 | IV | 423 | 0 |
| PL-25 | IIIB | 272 | 0 | PL-51 | IV | 424 | 1 |
| PL-26 | IV | 285 | 1 | PL-52 | IV | 428 | 1 |

**Clinical and pathological features of NSCLC patients for clinical lipid data**

| Sample ID | Stages | OS (days) | Survival state | Sample ID | Stages | OS (days) | Survival state |
| --- | --- | --- | --- | --- | --- | --- | --- |
| PL-53 | IV | 432 | 1 | PL-81 | IV | 756 | 0 |
| PL-54 | IV | 440 | 0 | PL-82 | IV | 760 | 1 |
| PL-55 | IIIB | 442 | 0 | PL-83 | IIIC | 761 | 1 |
| PL-56 | IV | 461 | 0 | PL-84 | IV | 771 | 0 |
| PL-57 | IV | 464 | 0 | PL-85 | IV | 780 | 0 |
| PL-58 | IIIC | 481 | 0 | PL-86 | IV | 808 | 0 |
| PL-59 | IV | 484 | 0 | PL-87 | IV | 813 | 0 |
| PL-60 | IV | 498 | 0 | PL-88 | IIIB | 818 | 0 |
| PL-61 | IV | 501 | 0 | PL-89 | IV | 827 | 1 |
| PL-62 | IV | 501 | 0 | PL-90 | IV | 835 | 0 |
| PL-63 | IIIB | 508 | 0 | PL-91 | IV | 850 | 0 |
| PL-64 | IIIB | 531 | 1 | PL-92 | IV | 854 | 0 |
| PL-65 | IV | 550 | 0 | PL-93 | IV | 918 | 0 |
| PL-66 | IV | 559 | 0 | PL-94 | IV | 932 | 0 |
| PL-67 | IV | 581 | 0 | PL-95 | IV | 940 | 0 |
| PL-68 | IV | 589 | 1 | PL-96 | IV | 952 | 1 |
| PL-69 | IV | 595 | 0 | PL-97 | IV | 955 | 0 |
| PL-70 | IV | 596 | 0 | PL-98 | IV | 974 | 0 |
| PL-71 | IIIB | 608 | 1 | PL-99 | IV | 986 | 0 |
| PL-72 | IV | 623 | 0 | PL-100 | IIIb | 1005 | 0 |
| PL-73 | IV | 626 | 1 | PL-101 | IV | 1020 | 0 |
| PL-74 | IV | 639 | 1 | PL-102 | IV | 1043 | 0 |
| PL-75 | IIIC | 641 | 0 | PL-103 | IV | 1052 | 0 |
| PL-76 | IV | 644 | 1 | PL-104 | IIIB | 1054 | 0 |
| PL-77 | IVA | 668 | 1 | PL-105 | IIIB | 1110 | 1 |
| PL-78 | IV | 700 | 1 | PL-106 | IIIB | 1130 | 0 |
| PL-79 | IV | 702 | 1 | PL-107 | IIIC | 1135 | 0 |
| PL-80 | IV | 706 | 1 | PL-108 | IV | 1147 | 0 |

| Sample ID | Stages | OS (days) | Survival state | Sample ID | Stages | OS (days) | Survival state |
| --- | --- | --- | --- | --- | --- | --- | --- |
| PL-109 | IV | 1151 | 0 | PL-131 | IV | 1508 | 0 |
| PL-110 | IV | 1162 | 0 | PL-132 | IIIC | 1508 | 0 |
| PL-111 | IIIC | 1164 | 1 | PL-133 | IV | 1510 | 0 |
| PL-112 | IV | 1170 | 1 | PL-134 | IIIC | 1515 | 0 |
| PL-113 | IV | 1180 | 0 | PL-135 | IVB | 1531 | 0 |
| PL-114 | IV | 1198 | 1 | PL-136 | IIIC | 1537 | 0 |
| PL-115 | IIIB | 1205 | 0 | PL-137 | IV | 1548 | 0 |
| PL-116 | IV | 1242 | 0 | PL-138 | IV | 1552 | 0 |
| PL-117 | IVA | 1279 | 0 | PL-139 | IV | 1567 | 0 |
| PL-118 | IV | 1297 | 0 | PL-140 | IV | 1606 | 0 |
| PL-119 | IV | 1303 | 0 | PL-141 | IV | 1622 | 0 |
| PL-120 | IV | 1314 | 0 | PL-142 | IV | 1667 | 0 |
| PL-121 | IIIb | 1319 | 0 | PL-143 | IV | 1725 | 0 |
| PL-122 | IV | 1337 | 0 | PL-144 | IV | 1734 | 0 |
| PL-123 | IIIB | 1344 | 0 | PL-145 | IV | 1739 | 0 |
| PL-124 | IV | 1347 | 0 | PL-146 | IV | 1751 | 0 |
| PL-125 | IV | 1361 | 0 | PL-147 | IV | 1797 | 0 |
| PL-126 | IV | 1388 | 0 | PL-148 | IIIB | 1834 | 0 |
| PL-127 | IV | 1414 | 0 | PL-149 | IIIB | 1898 | 0 |
| PL-128 | IV | 1435 | 0 | PL-150 | IV | 2012 | 0 |
| PL-129 | IIIB | 1447 | 0 | PL-151 | IV | 2143 | 0 |
| PL-130 | IV | 1486 | 0 |  |  |  |  |

*In the “Survival state” column, 1 indicates deceased and 0 indicates alive.

**Supplementary Table 4：**

**Probes or primers sequence used in the study**

| **qRT-PCR (5’-3’)** | | |
| --- | --- | --- |
| GAPDH | Forward  Reverse | CCTCCCGCTTCGCTCTCT  TGGCGACGCAAAAGAAGAT |
| CETP | Forward  Reverse | TCCCGCATGCTGTACTTCTG  TCTCCAGCACTGCCTTGAAC |
| CTBP1 | Forward  Reverse | TCGGAACCCTTCAGCTTTAGC  CTCGCTGTACCATGCAGCA |
| **shRNAs** | | |
| shCTBP1 | shRNA-1  shRNA-2 | CGTCCTCTTCTATGATCCATA  CGGGTTTGACAATATCGACAT |
| siCTBP1-1 | Sense  Antisense | GGACCUGGAGAAGUUCAAAGC  GCUUUGAACUUCUCCAGGUCC |
| siCTBP1-2 | Sense  Antisense | GACCUGGAGAAGUUCAAACC  GGCUUUGAACUUCUCCAGGUC |
| shCETP | shRNA-1  shRNA-2 | GCCAAGTCAAGTATGGGTTGC  GCCATTGACCTCCAGATCAAC |

| NSCLC tumor tissues | | | | | |
| --- | --- | --- | --- | --- | --- |
| Sample ID | TNM | Stages | Sample ID | TNM | Stages |
| #1-LUAD | pT1cN0M0 | IA3 | #5-LUAD | pT2bN0M0 | IIA |
| #2-LUAD | pT1bN0M0 | IA2 | #6-LUAD | pT3N0M0 | IIB |
| #3-LUAD | pT2aN0M0 | IB | #7-LUSC | pT3N1M0 | IIIA |
| #4-LUAD | pT1bN0M0 | IA2 | #8-LUSC | pT1cN0M0 | IA3 |

**Supplementary Table 5：**

**Baseline clinical and pathological characteristics of NSCLC patient-derived tumor tissues utilized for organoid establishment.**
